# Detonation Nanodiamonds: A Comparison Study by Photoacoustic, Diffuse Reflectance, and Attenuated Total Reflection FTIR Spectroscopies

**DOI:** 10.3390/nano10122501

**Published:** 2020-12-13

**Authors:** Dmitry S. Volkov, Petr K. Krivoshein, Mikhail A. Proskurnin

**Affiliations:** Chemistry Department, Lomonosov Moscow State University, Moscow 119991, Russia; pkrivoshein@gmail.com

**Keywords:** detonation nanodiamonds, photoacoustic IR spectroscopy, diffuse-reflectance IR spectroscopy, attenuated total internal reflection IR spectroscopy

## Abstract

The qualitative analysis of nanodiamonds by FTIR spectrometry as photoacoustic (FTIR–PAS), diffuse-reflectance (DRIFT), and attenuated total reflection (ATR) modalities was evaluated for rapid and nondestructive analysis and comparison of nanodiamonds. The reproducibility and signal-gathering depth of spectra was compared. The assignment of characteristic bands showed that only six groups of bands were present in spectra of all the modalities with appropriate sensitivity: 1760 (C=O stretch, isolated carboxyl groups); 1640–1632 (H–O–H bend, liquid water); 1400–1370 (non-carboxyl C–O–H in-plane bend and CH_2_ deformation); 1103 (non-carboxyl C–O stretch); 1060 (in-plane C–H bend, non-aromatic hydrocarbons and carbohydrates); 940 cm^−1^ (out-of-plane carboxyl C–O–H bend). DRIFT provides the maximum number of bands and is capable of measuring hydrogen-bonded bands and CH_x_ groups. ATR provides the good sensitivity for water and C–H/C–C bands in the range 2000–400 cm^−1^. FTIR–PAS reveals less bands than DRIFT but more intense bands than ATR–FTIR and shows the maximum sensitivity for absorption bands that do not appear in ATR-IR spectra and are expedient for supporting either DRIFT or FTIR–PAS along with depth-profiling. Thus, all three modalities are required for the full characterization of nanodiamonds surface functional groups.

## 1. Introduction

Nanodiamonds (ND), due to the nanometer size of their primary particles, have a surface with a significant area and many functional groups [1,2]. During the production cycle, the initial charge goes through several stages of chemical treatment, mainly of an oxidizing nature. This is necessary primarily for the removal of non-diamond forms of carbon. As a result, various functional groups form at the surface of nanodiamonds, which are responsible for the use of nanodiamonds as sorbents [1,3,4,5] and are crucial for technological and biomedical applications of nanodiamonds [6,7,8,9,10,11].

FTIR spectroscopy, usually in the mid-IR region, is widely used to characterize nanodiamonds and their surface groups [12]. Previous articles on the surface groups of nanodiamonds by FTIR are mostly based on sample-pellet transmission FTIR techniques [4]; less common are diffuse reflectance FTIR (DRIFT) [13] or attenuated total reflectance FTIR (ATR–FTIR) [14,15]. However, there are several contradictions in the selection and assignment of the main absorption bands of nanodiamonds, a unified scheme of sample preparation for the analysis of nanodiamonds has not been proposed, and rather wide comparative data on different samples or brands of nanodiamonds do not exist.

Transmission FTIR methods with pellets work with several milligrams of nanodiamond sample and ca. 150 mg of KBr or KCl, which are selected for the implementation of the Bouguer–Lambert–Beer law. The distribution of KBr particles and the test object should be uniform for better comparison between measurements [16]. Another disadvantage is the high hygroscopicity of KBr and KCl and the possible reactions of alkali metal halides with surface groups of nanodiamonds, the probability of which increases with grinding-assisted mechanochemistry of nanodiamonds with alkali metal halides [14]. KBr also absorbs radiation in the far infrared region, which does not allow for measurements below 400 cm^−1^ [17]; KBr or KCl should also be of an acceptable purity.

DRIFT is not the most common method for studying the surface of nanodiamonds due to the sensitivity to the particle distribution in the sample holder, the width of the nanodiamonds fraction; these features lead to low reproducibility of this method [13,18]. As transmission techniques, DRIFT requires the dilution of the test nanodiamond sample with KBr or KCl and subsequent grinding.

ATR–FTIR spectrometry is considered the most convenient selection for ND surfaces due to its simplicity and as it provides spectra comparable with the transmission spectra [7,19,20]. It does not require the sample dilution, which opens the far IR region. This method also allows for varying the depth of radiation penetration into the sample by changing the incidence angle of the radiation, but this feature is not implemented in most ATR units [21]. Additionally, it is considered that ND films and powders have the optimum particle size to achieve a good covering of the ATR crystal surface and sufficient adhesion. However, ATR–FTIR has several limitations. This method requires full contact of the test sample with the ATR crystal to improve the signal-to-noise ratio [16]. As diamond is one of the hardest known materials, the good contact between the NDs and the ATR crystal is a problem: materials such as ZnSe, Ge, or KRS-5 are too soft and their surface at a considerable pressure of the ATR press can be damaged. In addition, a part of the ND sample may be retained in the crystal, leading to fast degradation of the crystal operability. Thus, for NDs, only a diamond ATR crystal is applicable, but it does not allow for measuring the diamond itself, thus, the absorption of surface ND groups may be acquired. The recording of the ATR-IR spectra of nanodiamonds without using a clamping screw are described [22]: a colloidal solution of nanodiamonds is prepared in a highly volatile solvent placed to the crystal after drying and ATR–FTIR spectra are recorded. In addition, raw ATR–FTIR spectra are usually strongly distorted: shifts to lower wavenumbers are observed. This requires extra (extended) ATR correction [16], but the parameters for this mathematical transform are selected manually, often empirically; therefore, some information may be degraded or warped.

Interferometer-based FTIR photoacoustic spectroscopy (FTIR–PAS) is a developed instrument for complex samples [23,24,25,26,27]. It can be used for mineral, polymer, and technological samples. Some capabilities of FTIR–PAS are analogous to ATR–FTIR, and some to DRIFT. As ATR–FTIR, it does not need sample dilution and is applicable in the far infrared (IR) region. FTIR–PAS has some advantages. It allows for varying the depth of radiation penetration by changing the modulation frequency, which provides a longer range of depths. FTIR–PAS was used to crush diamonds down to a micron size [28], FTIR–PAS and DRIFT methods showed comparable results [28]. However, as far as we know, the FTIR–PAS method has not been used to date for studying the surface of nanodiamonds. The whole comparison of various modalities for complex samples (biochars, soils) has recently become a topic of several review or feature papers [29,30]; however, usually the research is focused on specific characteristics, such as depth profiling in FTIR–PAS [27,31,32]. For nanodiamonds, as far as we are concerned, a full comparison of FTIR–PAS, DRIFT and ATR features in spectral information was not done.

The growing number of studies dealing with various modifications in the nanodiamond surface makes serious demands of the methods of surface characterization. Thus, the aim of this work was to compare the spectral information provided by these modalities for the same samples measured under the same conditions. To assess their capabilities from the point of view of maximum information content and reliability of the spectral information provided, we studied the surface of NDs using ATR–FTIR, DRIFT, and FTIR–PAS and compared the results for a rather wide selection of various ND brands.

## 2. Materials and Methods

### 2.1. Nanodiamonds

We used commercially available NDs, summed up in Table 1. Powders from aqueous suspension (SDND, PL-Nanopure-G01P) stock solutions were prepared by drying in a drying oven (SNOL 20/300, Snol-Therm, Russia) at 100 °C. For analysis, nanodiamond powder was used as is or nanodiamonds were gently ground in a jasper mortar.

### 2.2. Instrumentation

IR spectra of nanodiamond powders were recorded on a Bruker Vertex 70 single-beam IR Fourier spectrometer (Bruker Optik GmbH, Ettlingen, Germany) equipped with a KBr beam splitter and a wide-range room temperature DLaTGS detector or liquid nitrogen cooled photovoltaic MCT detector. The spectrometer and accessories were continuously purged with −70 °C dew point air (produced by a PG28L Purge Gas Generator, PEAK Scientific, Glasgow, UK) with a flow of 500 L/h. The overall laboratory temperature was maintained at 23 °C with an allowable variation in ±1 °C using an air conditioner.

#### 2.2.1. FTIR–PAS

An MTEC PAC300 photoacoustic accessory (MTEC Photoacoustic, Inc., Ames, IA, USA) and FTIR–PAS spectra were obtained by varying the scanning frequency; software correction of the peaks of CO_2_ and H_2_O was not used. The parameters for recording FTIR–PAS spectra are shown in Table 2. Samples of nanodiamonds were placed in a cell that was installed in the accessory, the cell compartment was purged with helium for 5–10 s. The number of scans for a sample and background: at an interferometer frequency of 1.6 kHz, this is 64 scans; at 2.5 kHz this is 128 scans; at 5 kHz, this is 256 scans. In FTIR–PAS mode, before each image, the spectrum of the background signal was recorded using highly pure compressed graphite. Samples weighing 5–10 mg were examined. For interferometer frequencies of 1.6 and 2.5 kHz, sample signal amplification modes were 1000 and 2000 times, respectively.

The resulting spectra were subjected to automatic baseline correction and 25-point smoothing.

#### 2.2.2. ATR–FTIR

The GladiATR^TM^ single reflection attenuated total internal reflection accessory with a diamond crystal (Pike Technologies, Madison, WI, USA) was used for spectra registration in ATR mode. The accessory was configured for heating up to 215 °C. A background signal was recorded prior each new sample. The registration parameters of ATR spectra are shown in Table 3. Samples of nanodiamonds were transferred in a dry state to a crystal of an ATR attachment, and then clamped with a clamping screw.

Room temperature DLaTGS did not have a software-variable gain level; therefore, in all measurements, the “Ref” level was used, which means no gain in OPUS software. The Photovoltaic MCT DETECTOR had three gain levels in order of increasing gain: no gain (“Ref”), a standard (low) gain (A), and a medium gain (B).

Before recording the spectra with heating, the spectrum of an empty ATR crystal was recorded at 25 °C as a background, then a small amount of the sample was placed on its surface, pressed with a screw, and a program of controlled heating was started at a rate of 0.25 °C/min from 25 °C to 215 °C, and spectra registration step every 2.5 °C. After heating to 215 °C and recording the spectrum, the sample was cooled to 25 °C. The resulting array of heating spectra (25–215 °C) was combined, and then a similar array of spectra of an empty ATR crystal was subtracted from it.

#### 2.2.3. DRIFT

The PrayingMantis^TM^ diffuse reflection accessory (Harrick Scientific Products, INC, Pleasantville, NY, USA) was used for spectra registration in diffuse reflectance mode. The registration parameters of DRIFT spectra are shown in Table 4.

Software automatically converted spectra measured in diffuse reflectance using the Kubelka–Munk (KM) conversion. The conversion is based on the following formula:

(1) $$KM=\frac{{\left(1-Refl\right)}^{2}}{\left(2\times Refl\right)}$$

where Refl is the relation between reflected and incident light. Absorbance spectra are first converted to transmittance spectra. The smallest value allowed for transmittance or reflectance is 0.001%. This equals a Kubelka–Munk value of about 500.

For background measurements the tilted alignment mirror for PrayingMantis^TM^ accessory was used. For this, the alignment fixture was slid into the accessory, with the horizontal mirror going in first. In this orientation, the tilted mirror is in the sampling position. We compared this approach with KBr in the sample cup.

### 2.3. Data Handling

All spectra were processed in OPUS 7.5 software (Bruker Optik GmbH, Ettlingen, Germany). FTIR–PAS spectra were smoothed out by 25 points, and DRIFT and ATR–FTIR by 13 points (at some figures, spectra are presented without smoothing).

The penetration depth of damped IR radiation with the wavenumber $\tilde{v}$ into the sample (approaching the ATR crystal/sample boundary at an angle θ to the normal) was estimated as:

(2) $$\mu_{ATR}\left(\tilde{v}\right)={\left(2\pi \tilde{v}n_{ATR}\sqrt{\left(\sin\theta^{2}-{\left(n_{ATR}/n_{S}\right)}^{2}\right)}\right)}^{-1}$$

where $n_{ATR}$ and $n_{S}$ are refractive indexes of the ATR crystal and sample, respectively.

In FTIR–PAS, the estimation of the radiation penetration depth is made using the dependence of the penetration depth of the heat wave

(3) $$\mu_{PAS}\left(\tilde{v}\right)=\sqrt{D_{T}/2\pi V\tilde{v}}$$

where *D_T_* is the thermal diffusivity of the sample and *V* is the interferometer mirror velocity [32]. Thermal diffusivity *D_T_* was taken as 7.8 cm^2^/s [33], the velocities of the difference in optical paths of the IR spectrometer were obtained from the interferometer modulation frequencies and the He–Ne laser reference lines were 0.1012 and 0.1582 cm/s, respectively.

### 2.4. Procedures

The FTIR–PAS spectra obtained by method 3 with an interferometer frequency of 1.6 kHz and an amplification of the signal of sample 1000 were smoothed over 25 points and the intensity was multiplied by $\sqrt{\tilde{v}}$ [32]. The ATR spectra were additionally transformed as follows: *I*_0_*a + b = I*_corr_, where *I*_0_ is the intensity of the ATR spectra after the operations described above, *I*_corr_ is the intensity of the ATR spectra for comparison with the FTIR–PAS spectra, *a* = 150, *b* = 1.

#### Reproducibility

To compare reproducibility, ten ATR-IR spectra of nanodiamonds were recorded with reregistration of the background spectrum after each measurement due to the high abrasive ability of nanodiamonds. After registration, the spectra were not subjected to extended ATR correction. Baseline correction and anti-aliasing were not applied.

10 FTIR–PAS spectra were recorded according to method 3 with a complete overfilling of the sample cup and an interferometer frequency of 1.6 kHz. FTIR–PAS spectra were not subjected to automatic baseline correction and smoothing. RSD and signal-to-noise ratio (S/N=1/RSD) were calculated as

(4) $$\mathrm{RSD}=\sqrt{\frac{\sum_{i}{\left(x_{i}-\bar{x}\right)}^{2}}{n-1}}\frac{1}{\bar{x}}$$

## 3. Results

### 3.1. Band Assignment

**Figure 1:**
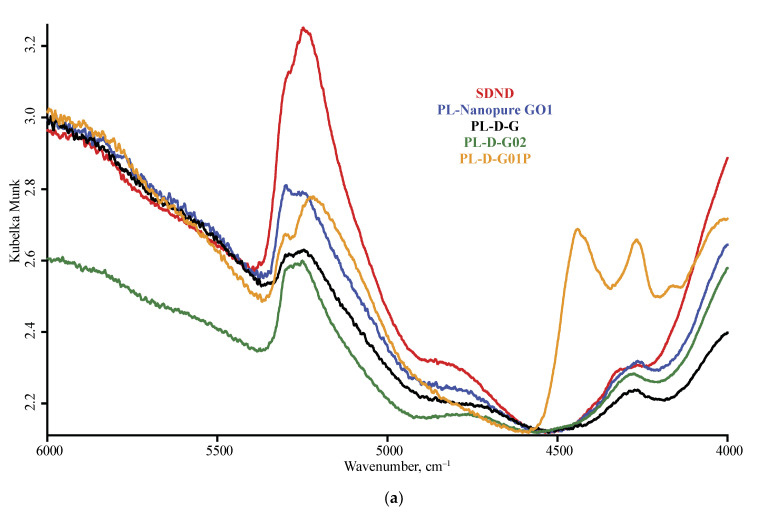

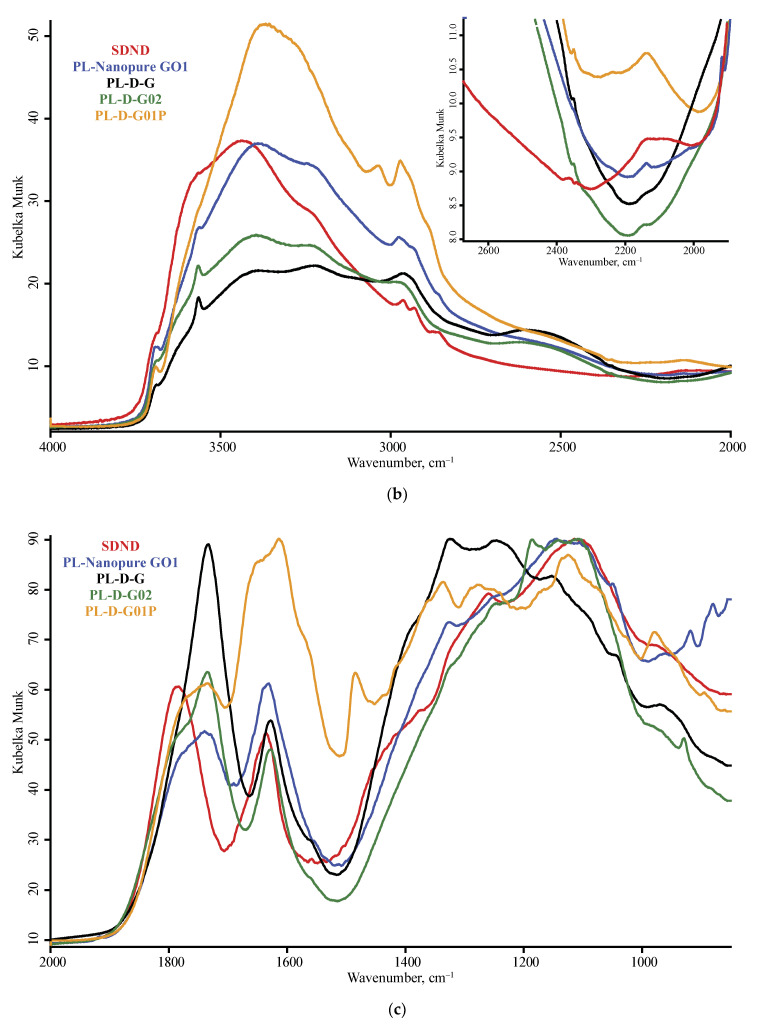
Diffuse reflectance mode, LN-MCT photovoltaic detector; nanodiamond brands: red, SDND; blue, PL-Nanopure GO1; black, PL-D-G; green, PL-D-G02; orange, PL-D-G01P; (**a**) 6000–4000 cm^−1^, (**b**) 4000–2000 cm^−1^ (**c**) 2000–800 cm^−1^ range. Spectra are smoothed (13 points) and normalized to maximize each spectrum.

**Figure 2:**
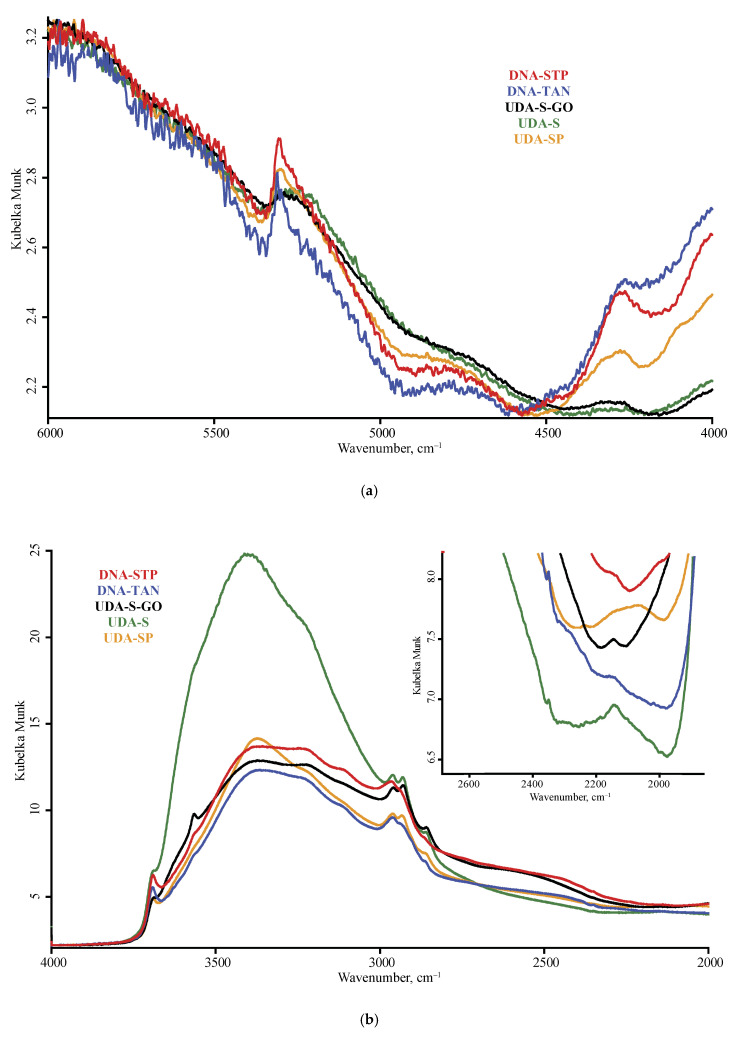

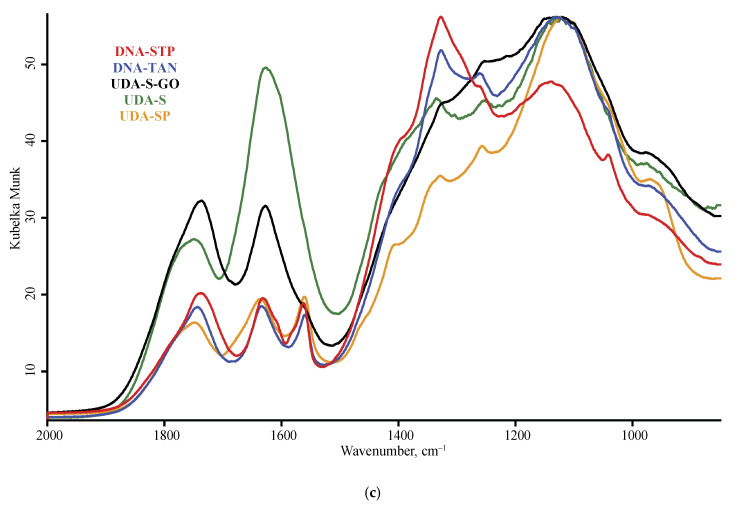
Diffuse reflectance mode, LN-MCT photovoltaic detector; nanodiamond brands: red, DNA-STP; blue, DNA-TAN; black, UDA-S-GO; green, UDA-S; orange, UDA-SP; (**a**) 6000–4000 cm^−1^, (**b**) 4000–2000 cm^−1^ (**c**) 2000–800 cm^−1^ range. Spectra are smoothed (13 points) and normalized to maximize each spectrum.

**Figure 3:**
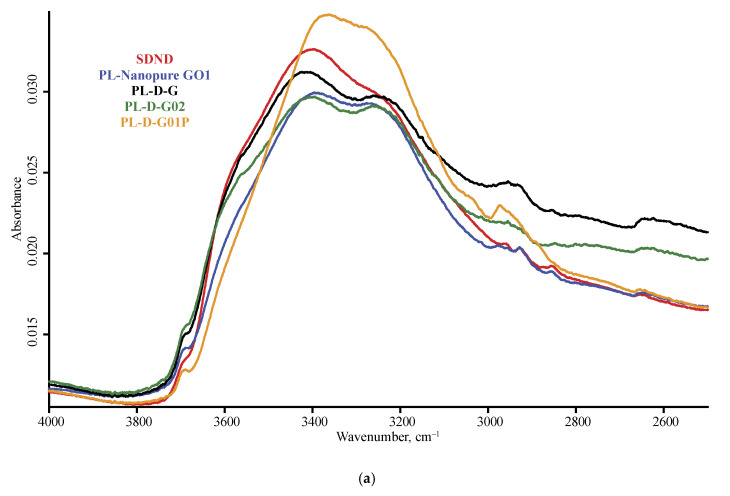

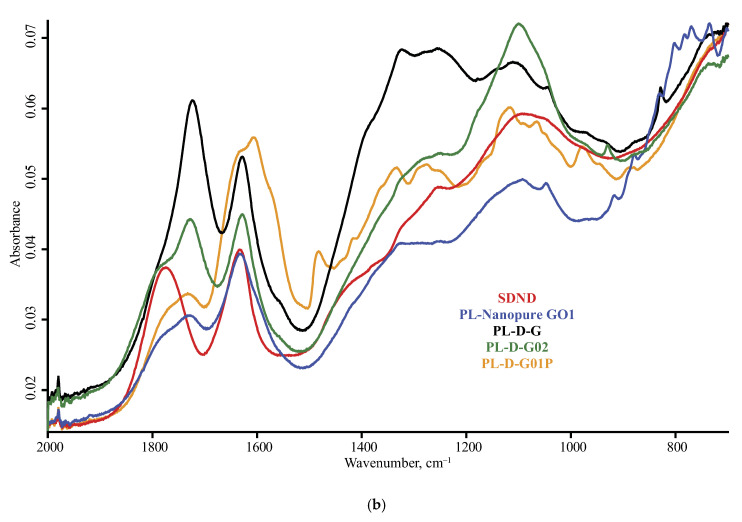
ATR mode, LN-MCT photovoltaic detector; nanodiamond brands: red, SDND; blue, PL-Nanopure GO1; black, PL-D-G; green, PL-D-G02; orange, PL-D-G01P; (**a**) 4000–2500 cm^−1^, (**b**) 2000–400 cm^−1^ range. Spectra were smoothed (13 points) and normalized to maximize each spectrum.

**Figure 4:**
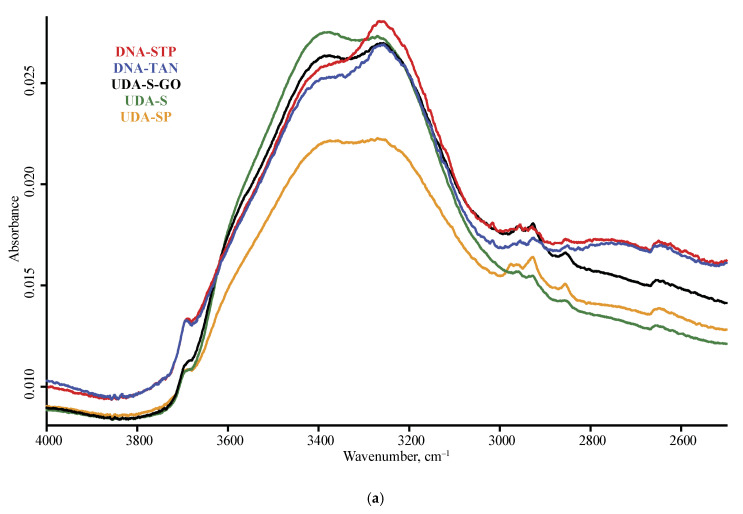

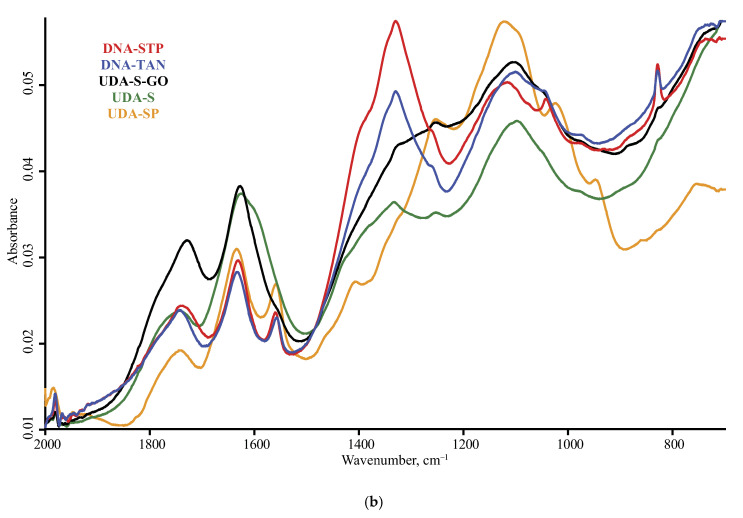
ATR mode, LN-MCT photovoltaic detector; nanodiamond brands: red, DNA-STP; blue, DNA-TAN; black, UDA-S-GO; green, UDA-S; orange, UDA-SP; (**a**) 4000–2500 cm^−1^, (**b**) 2000–400 cm^−1^ range. Spectra were smoothed (13 points) and normalized to maximize each spectrum.

**Figure 5:**
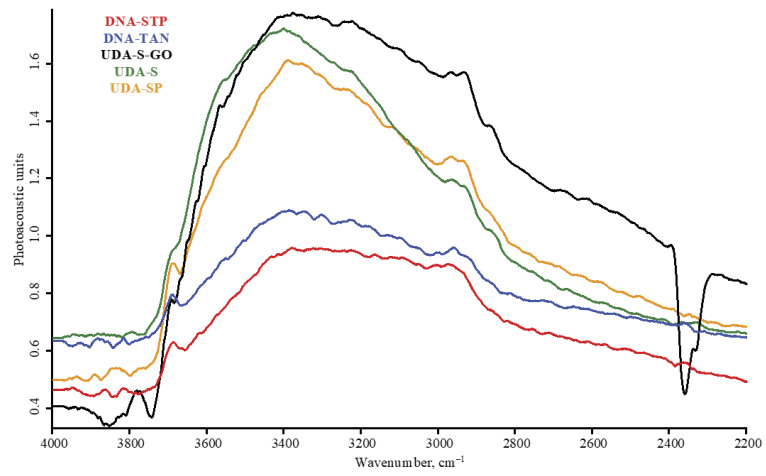

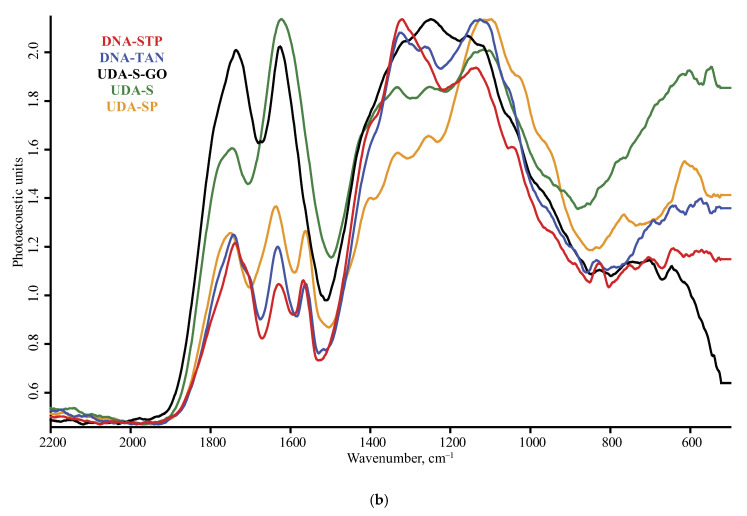
Photoacoustic mode, 1.6 kHz; nanodiamond brands: red, DNA-STP; blue, DNA-TAN; black, UDA-S-GO; green, UDA-S; orange, UDA-SP; (**a**) 4000–2200 cm^−1^, (**b**) 2200–400 cm^−1^ range. Spectra were smoothed (25 points) and normalized to maximize each spectrum.

**Figure A1:**
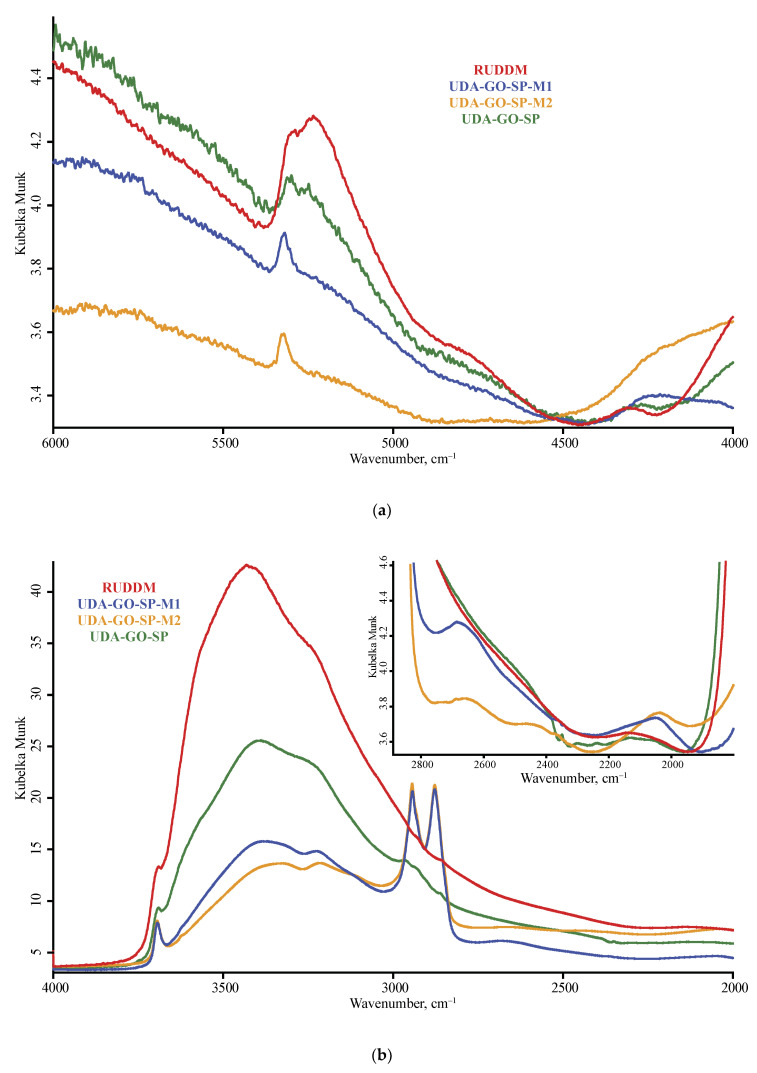

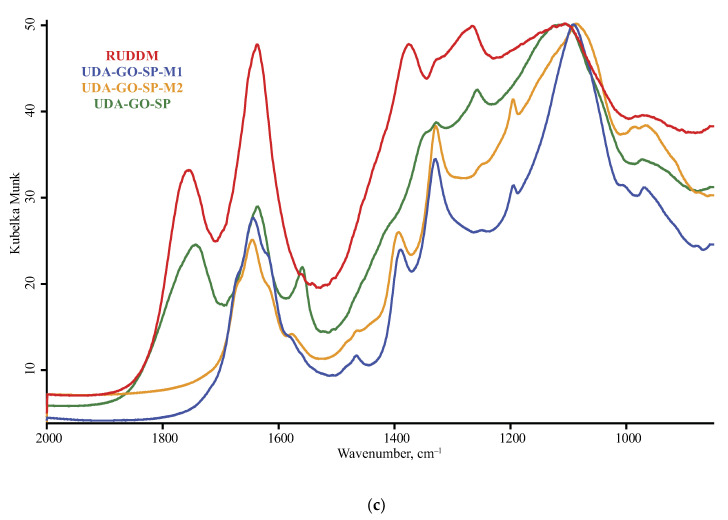
Diffuse reflectance mode, LN-MCT photovoltaic detector; nanodiamond brands: red, RUDDM; blue, UDA-GO-SP-M1; orange- UDA-GO-SP-M2; green, UDA-GO-SP; (**a**) 6000–4000 cm^−1^, (**b**) 4000–2000 cm^−1^ (**c**) 2000–800 cm^–1^ range. Spectra were smoothed (13 points) and normalized to maximize each spectrum.

**Figure A2:**
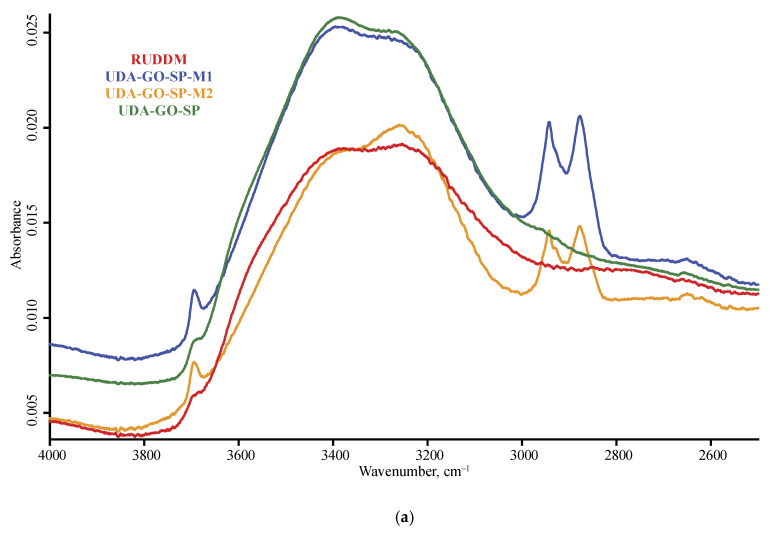

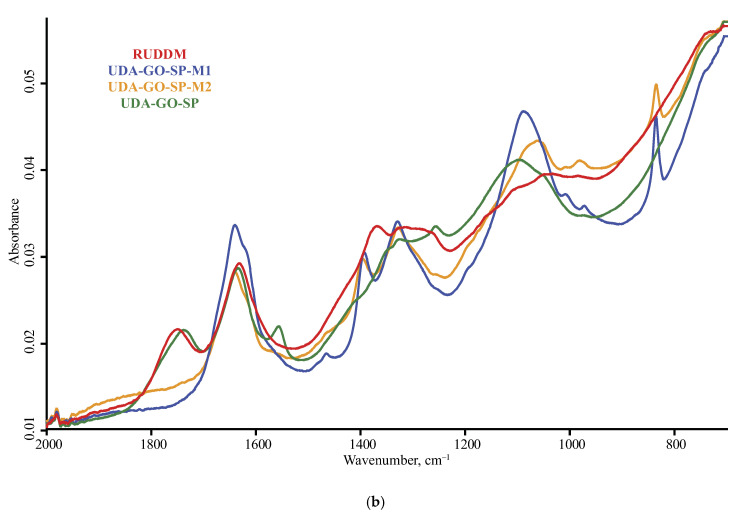
ATR mode, LN-MCT photovoltaic detector; nanodiamond brands: red, RUDDM; blue, UDA-GO-SP-M1; orange, UDA-GO-SP-M2; green, UDA-GO-SP; (**a**) 4000–2500 cm^−1^, (**b**) 2000–400 cm^−1^ range. Spectra were smoothed (13 points) and normalized to maximize each spectrum.

The spectra of nanodiamonds for the studied brands are shown in Figure 1 and Figure 2, and Figure A1 (Appendix A) for DRIFT, Figure 3 and Figure 4, and Figure A2 for ATR, and Figure 5 for FTIR–PAS. All the assigned bands existing in the spectra of all the selected ND brands are summed up in Table 5. In this section, we describe the assignment of all major bands present in most brands in at least two modalities and all the measurement conditions; the comparison and differences in spectra for IR modalities and specific features of brands are given in the next section. 

The near IR region shows two wide bands at 5900–5600 and 4300 cm^−1^ that are C–H first overtone and combination bands, respectively (Figure 1a). In some cases, a weak broad band at 4800 cm^−1^ that may be attributed to aromatic C–H combination bands is present. They are revealed by DRIFT and FTIR–PAS and not ATR. Additionally, these modalities show a rather explicit combination band of water at 5300 cm^−1^ with intensities correlated with the intensities of water bands in the mid-IR range.

In the shortwave mid-IR region, a wide band of 3700–3000 cm^−1^ of O–H stretch and intermolecular hydrogen bonds [35] and, at its shoulder, sharp bands at 3695 cm^−1^ of isolated water and 3569 cm^−1^ attributed to hydrogen-bon bonded water and non-water –OH groups are observed [34,35,41]. These bands are present in DRIFT and FTIR–PAS and are not reliably detected with ATR (Figure 3 and Figure 4). Some brands show another sharp band at 3715 cm^−1^ that can be attributed to ROH···HOR hydrogen bonds.

Bands of O–H stretching vibrations (3500–3200 cm^−1^) are present in all the spectra, and the shape depends on the modality and ND brand, the maxima of asymmetric and symmetric bands at 3410 and 3230 cm^−1^ correspond to strongly hydrogen-bonded species and, in several cases, the band at 3290 cm^–1^, which is probably the overtone band of liquid adsorbed water, 2*v*_2_ is present [16].

All four asymmetric and antisymmetric aliphatic vibrations of sp^3^ CH_x_ stretch vibrations in the range 3100–2800 cm^−1^ are observed, and in most brands, ATR shows low sensitivity (Table 5). The band at 1470 cm^−1^ corresponds to CH_2_ wagging and correlates with the intensity of CH_x_ stretch at the 2900 cm^−1^ range. Bands at 1400–1395 and 1375–1370 cm^−1^ can be attributed as CH_2_/CH_3_ deformation bends as well as C–O–H bends (see below), so their intensities are not correlated with purely CH_x_ bands at 3100–2800 cm^−1^.

Apart from aliphatic bands, some brands show aromatic C–H stretching at 3100–3050 cm^−1^ [42] and the band at 1580–1560 cm^−1^ (most probably, C=C stretch). These bands are accompanied with distinct bands at 1060 and 760 cm^−1^ that can be attributed to in-plane C=C–H and polyaromatic C=C–H bending, respectively. Additionally, a band of 410 cm^−1^ is attributed to C–C in-phase vibrations [40]. A band at 960 cm^−1^ can be attributed to =CH_2_ wag.

**Figure 6:**
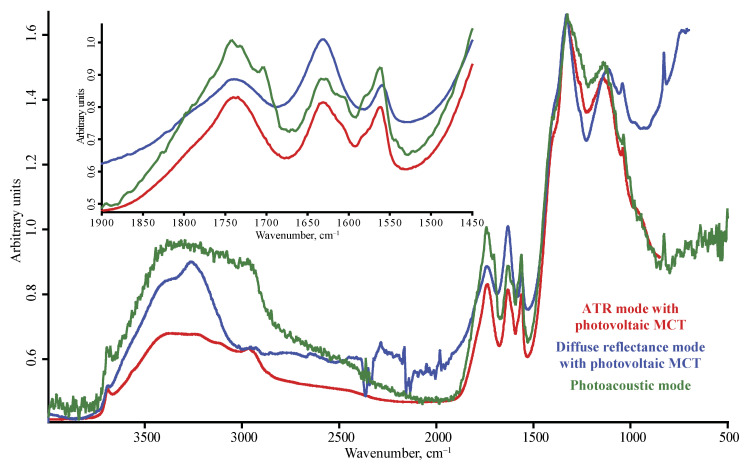
DNA-STP nanodiamonds; red, ATR mode with photovoltaic MCT detector; blue, diffuse reflectance mode with photovoltaic MCT detector; green, photoacoustic mode. Spectra are not smoothed but normalized to maximize each spectrum.

The range of 1900–1200 cm^−1^ reveals the maximum number of bands. Here, the stretch of the carbonyl C=O (1770–1650 cm^−1^) is dominant, usually in the form of a continuum from 2000 to 1600 cm^−1^; bands at 1760–1740 cm^−1^ attributed to the carbonyl of carboxylic groups and less strong bands (for not all the brands) at 1670 cm^−1^ attributed to the C=O stretch of non-carboxyl moieties are dominant (Figure 6). There is also a weak broad band of 1550 cm^−1^, also belonging to asymmetric C=O stretching vibrations [35,43] or asymmetric stretching vibrations of deprotonated carboxyl O–C=O [43,44]; however, the comparison of the blank in FTIR–PAS, ATR, and DRIFT shows that such low-intensity bands in the range 1600–1400 cm^−1^ are mostly enveloped narrow bands of gaseous water. In the case of DRIFT and ATR, they are positive, but in the case of FTIR–PAS, these peaks are inverted, and in the case of low-intensity, signals can be wrongly interpreted as the broader bands red-shifted, compared to other modalities. These artefact bands were excluded from further consideration.

The band intensities at 1700 cm^−1^ correlate well with the signal from a weak broad band 2700–2600 cm^−1^, which is attributed to O–H stretch in carboxyl, which is red-shifted O–H compared to the main continuum of 3500–3200 cm^−1^. The presence of this band can be considered as a proof of carboxylic groups at the surface.

**Figure 7:**
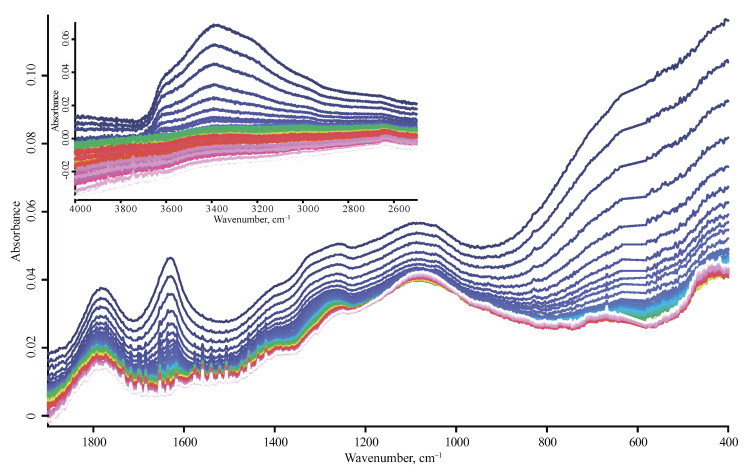
ATR mode, SDND nanodiamonds, heating from 25 °C (blue) to 215 °C (pink). Spectra are not smoothed.

**Figure A3:**
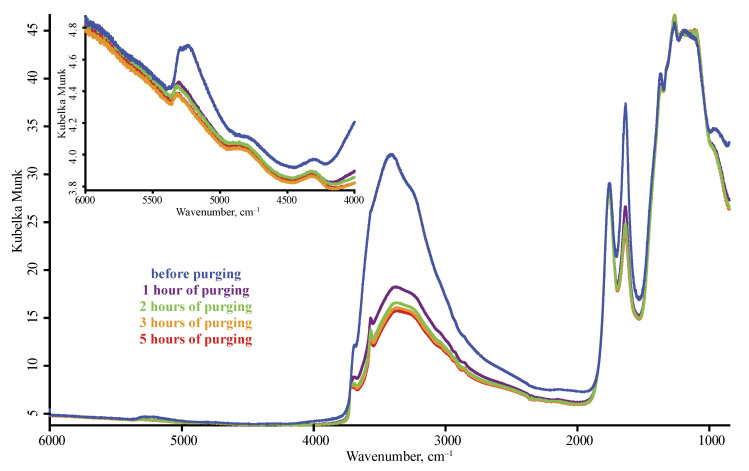
RUDDM nanodiamonds, DRIFT with MCT; blue, immediately when the sample was placed into accessory; purple, after 1 h of purging sample inside accessory; green, 2 h; orange, 3 h; red, 5 h. Spectra were not smoothed and normalized.

A band of H–O–H bending vibrations of liquid unbound or loosely bound water 1643 cm^−1^ is present in all the spectra; all the studied air-dried samples contain 5–7% of water [45,46]. Another band of water (1620–1635 cm^−1^) is present in all the spectra. Its wavenumber is red-shifted compared to liquid water (1643 cm^−1^), evidencing a high contribution of hydrogen-bonding to the vibrations [47]. Its intensity decreases after drying, but this band does not disappear even after five hours of drying, using ultra dry air at room temperature inside the DRIFT accessory (Figure A3, Appendix A). These bands almost disappeared at temperatures above 100 °C (Figure 7). The presence of this band is evidence of the water layer at the surface of the nanodiamonds that determines the colloidal solubility of nanodiamonds and its division into “soluble” and “insoluble” brands [5]. In this work, we used drying to confirm the correct assignment of water bands, especially at the 1700–1600 cm^−1^ region.

Symmetrical bands at 1440 and 1390–1320 cm^−1^, at least partly, can be assigned to C–O–H in-plane bend for carboxyl [37,43,44] and non-carboxyl groups [38]. The bands at 1270 cm^−1^ correspond to carboxyl C–O stretch or bending vibrations of epoxy C–O [35,43,44]. Weakly pronounced asymmetric bending vibrations that can be assigned to C–O–C are observed in the range of 1150–1110 cm^−1^ [35,43,44]. The band at 1100–1040 cm^−1^ can be attributed to non-carboxyl C–O stretch or asymmetric bending vibrations of C–O–C [35,42,43,44,48] and carboxyl and non-carboxyl out-of-plane C–O–H bend at 940 and 610 cm^−1^, respectively. The range of 1000–500 cm^−1^ has an increased overall intensity caused by the extra broad band of water librations. This mostly affects ATR and FTIR–PAS measurements.

### 3.2. Signal-Gathering Depth and FTIR–PAS Modulation Frequency Comparison

The refractive index of nanodiamonds is not equal to the refractive index of diamond due to the developed surface and the presence of many surface groups [16], but it is likely that the refractive index of nanodiamonds of any grade is within the value 2.4 ± 0.1 [49]. Thus, the estimation of the penetration (signal-gathering) depth by ATR with Equation (1) at 4000 cm^−1^ gives the value 240 nm, which is comparable to the characteristic size of ND clusters [43,50,51]. The maximum penetration depth of radiation in ATR measurements for a wavenumber of 400 cm^−1^ is ca. 2.4 μm, which is more than two orders of magnitude larger than the particle size of the studied nanodiamonds. This agrees with the sensitivity of ATR, which is lower than that of DRIFT and FTIR–PAS in the range 4000–2000 cm^−1^; additionally, the reproducibility of ATR in this range is also degraded compared to a low wavenumber range—see the next section.

**Figure A4:**
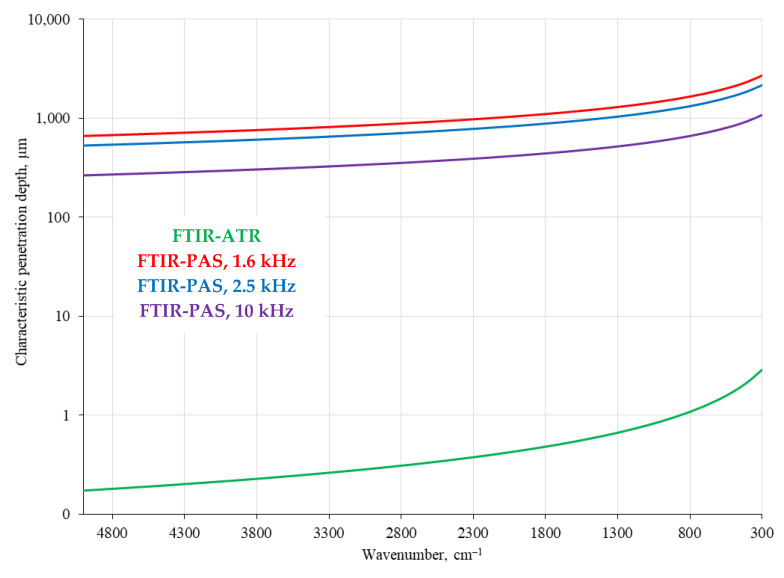
Radiation penetration depth into nanodiamond sample for ATR (green curve) and photoacoustic IR measurements at interferometer modulation frequencies 1.6 (red), 2.5 (blue) and 10 kHz (violet line).

To estimate the penetration depth of radiation by FTIR–PAS, we performed a model calculation of *μ*_s_ for interferometer modulation frequencies (IMF) of 1.6, 2.5, and 10 kHz using Equation (3), Figure A4 (Appendix A). The penetration depth of the radiation in all the mid-IR ranges is 300–1000 µm, which is much greater than the size of the investigated particles (0.1–0.2 µm). Therefore, the radiation in FTIR–PAS must completely pass through all particles and each individual particle must emit thermal waves in all directions of space and give a high signal to the detector [52].

The FTIR–PAS spectrum of nanodiamonds at 2.5 kHz has a large baseline profile in the range of 4000–1900 cm^−1^. The absorption bands of asymmetric 2920 cm^–1^ and symmetric 2860 cm^−1^ [42] stretching –CH_x_ vibrations remain in the spectra. The reason for the differences in spectra is the selection of the amplification mode for the sample signal. A twofold change in the signal amplification mode and a slight decrease in the radiation penetration depth causes significant changes in the spectrum. In both spectra, the band of stretching vibrations –C=O at 1750 cm^−1^ [44], and the band of stretching vibrations of the deprotonated carboxyl group at 1680 cm^−1^ [44] are manifested. There is also a band of asymmetric stretching vibrations of deprotonated carboxyl –C=O 1550 cm^−1^ [43,44]. Additionally, at 1.6 kHz, the band of asymmetric stretching vibrations of the carboxyl C–O stretch at 1270–1250 cm^−1^ and symmetric C–C–C vibrations with a wavenumber of 1190–1100 cm^−1^ [35,43,48] are weaker. The 970 cm^−1^ band of C–O–C bending vibrations [42] has a high intensity. FTIR–PAS spectra at an IMF of 5 kHz shows the same bands as lower IMF with the signal–noise ratio, thus with degraded information. A serious difference between low and high IMFs may result from thermal scattering that increases the overall background in FTIR–PAS. Thus, high IMFs for FTIR–PAS of nanodiamond powders are not recommended.

### 3.3. Band Reproducibility

For ATR–FTIR and DRIFT, the shape of the all the test bands and the positions of the maxima are reproduced, except for the 1750 cm^−1^ band (carboxyl C=O), where a small scatter is observed after vector normalization. The range of 3000–2000 cm^−1^, as well as the absorption bands of CH_x_ stretching vibrations, contain CO_2_ absorption bands and, in ATR, the absorption band of the ATR attachment crystal (2350–1900 cm^−1^), which seriously degrade the reproducibility of ATR measurements in this range. In the range of 2000–1000 cm^−1^, the band at 1400 cm^−1^ (C–O/C–H) has the highest RSD value among all the analyzed bands due to its lowest intensity in this range. In the range of 1000–400 cm^−1^, a significant increase in the baseline is observed due to water librations, which entails the need for a baseline-correction procedure. 

For FTIR–PAS (IMF, 1.6 kHz), the shapes and positions of band maxima are also reproduced. In the range of 4000–3000 cm^−1^, many narrow bands in the range 3920–3550 cm^−1^ are observed, which belong to stretching vibrations of adsorbed water either on the walls of the IR spectrometer and attachment on the surface of nanodiamonds. After the vector normalization procedure, main absorption bands at 1760 and 1630 cm^−1^, as well as 1267 cm^−1^ are most clearly traced. High-intensity artifacts are observed in the range 500–400 cm^−1^, which is why this range is not considered further in FTIR–PAS. For two spectra out of ten, artifacts are observed in the entire range over the entire considered range of wavenumbers. The bands at 1400 and 1750 cm^−1^ have the best RSD values. As shown in the previous section, the particle size of nanodiamonds is significantly lower than μ_ATR_ in the whole spectral range. However, decreasing the radiation penetration depth upon shifting from the IMF from 1.6 to 2.5 kHz lowers the intensities of all the characteristic bands and increases RSD values, thus negatively affecting the signal-to-noise ratio. Thus, to record a FTIR–PAS spectrum of nanodiamonds in the range 4000–2000 cm^−1^, it is preferable to use an IMF of 1.6 kHz with a 200-fold amplification of the background signal and a 1000-fold gain in the sample signal.

The measurement errors for mid-IR spectra can be summed up as follows (spectra reproducibility errors were found by Equation (3); spectra at this stage were not subjected to automatic baseline correction and smoothing). The RSD values of the integrals of the main bands (Table 5) are presented in Table 6, Table 7 and Table 8 for ATR, FTIR–PAS, and DRIFT, respectively. In the range 4000–3800 cm^−1^, the RSD can be up to 100% in FTIR–PAS; in ATR-IR and DRIFT, it does not exceed 35%. In 3800–3000 cm^−1^, the RSD is 15–30% for all the techniques. In 3000–2500 cm^−1^, the RSD for FTIR–PAS is also 15–40%; for ATR this is lower, at 18–20% (in the absorption range of the diamond crystal, 2350–1900 cm^−1^, this is 35–45%); for DRIFT this is 20–30%. In 1900–1000 cm^−1^, the minimum RSD in FTIR–PAS is 25%, and no more than 15–25% for ATR, and 12–20% for DRIFT. In 1000–400 cm^−1^, the RSD for FTIR–PAS and DRIFT does not exceed 40%; in ATR, this does not exceed 20%. Therefore, for NDs, DRIFT shows the best results; the second best is ATR, despite the possibility of mechanical damage to the diamond crystal of the ATR attachment. The spectra are well reproduced, the baseline is less distorted and stable, apart from in the 1000–400 cm^−1^ range, and no spectral artifacts appear in the whole interval. The same cannot be said for the FTIR–PAS spectra, showing some spectral artefacts. However, in general, the RSD profiles are similar; most differences are observed in the absorption range of the diamond crystal of the ATR attachment (2350–1900 cm^−1^). 

## 4. Discussion

### 4.1. DRIFT

This modality provides the maximum number of bands and shows the highest sensitivity in the whole range, from NIR to FIR. The bands discussed above in the NIR range above 4000 cm^−1^ are obtained only with this modality, due to a lower noise in DRIFT.

**Figure 8:**
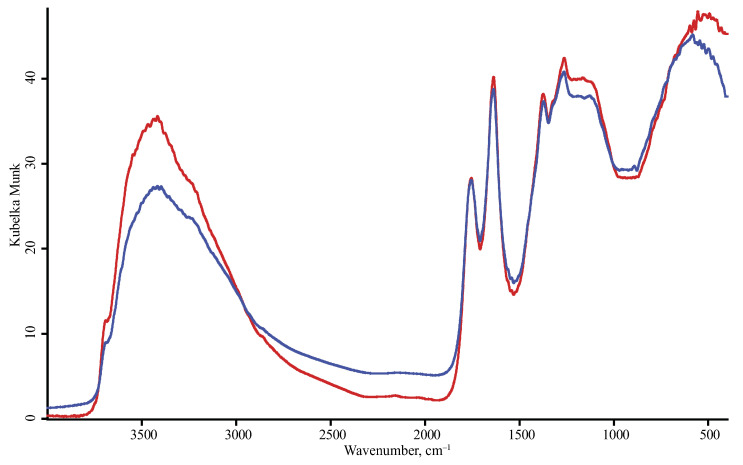
Diffuse reflectance mode, DLaTGS detector, RUDDM nanodiamonds; red, PrayingMantis mirror was used for background collection; blue, KBr in the sample cup was used for background collection. Spectra are smoothed (13 points) and normalized to maximize each spectrum.

In this study, we used DRIFT with a bare mirror as the background reference sample, the technique that is used for detector or spectrometer calibration [53] but more seldom used in practical DRIFT analysis. The used DRIFT attachment also allows diffuse-reflectance measurements without sample dilution with KBr, without a change in the reproducibility and sensitivity (Figure 8).

This modality is capable of hydrogen-bond continua at 3900–3000 and 2700–2500 cm^−1^. The latter range can be seen only with DRIFT, as ATR has not enough sensitivity, while FTIR–PAS shows a high-level noise. As shown in Section 3.3, this range suffers an increase in the noise level due to diamond crystal absorption. The range 2400–2300 always shows the artefact peaks of gaseous CO_2_.

DRIFT also provides the best resolution and sensitivity towards hydrocarbon constituents of the ND shell: CH_x_ groups at 3000–2700 cm^−1^. These are accompanied with CH_2_/CH_3_ deformation bends at 1400 and 1370 cm^−1^, respectively (usually as shoulder bands); however, these bands could also have oxygen-containing components. Additionally, this modality provides well resolved and intense bands at, 1450 cm^−1^ (C–O–H bend and –CH_2_ wag vibrations); thus, these ranges would require either a sample preparation or another modality.

Depending on the brand, the CH_x_ range in DRIFT shows stretch aromatic vibrations at 3100 cm^−1^ with rather high sensitivity and correlated bands 940 (=CH_2_ wag) and 830 cm^−1^ (aromatic –C–H bend), as shown in Figure 1c, Figure 3b and Figure 5b. Some brands show a weak band at 760 cm^−1^ (Figure 1c and Figure 3b) that can be attributed to polyaromatic bend vibrations, and the latter band is not seen neither in FTIR–PAS, nor in ATR modalities. Carbon–carbon bonds are represented by 1560 cm^−1^ (alkene/aromatic stretch) and 410 cm^−1^ (in-phase vibrations).

The carbonyl band at 1760 cm^−1^ is most intense in the range 1900–1300 cm^−1^ (Figure 1b and Figure 2b). The right shoulder band at 1790 cm^−1^ (conjugated carbonyl groups) is less pronounced compared to ATR and FTIR–PAS (DNA-TAN and DNA-STP brands).

The H–O–H band at 1610 cm^−1^, to which we assign more tightly bound water is weaker than in FTIR–PAS and especially ATR. This allows for revealing actual bands belonging to asymmetric C=O and C=C stretches in this area (PL-D-G01P, UDA-S, UDA-SP, and DNA-STP).

Despite most intense bands in the range of 1400–1200 cm^−1^, this is not well-resolved (Figure 1c and Figure 2c), most probably due to saturation effects [54], and this range is not expedient for band comparison.

Of special interest in DRIFT modality is the range 2300–2000 cm^−1^, which is not informative in ATR with a diamond crystal due to diamond adsorption and FTIR–PAS due to a high noise level. This range shows a series of rather broad and rather weak bands located at 2230, 2140, and 2050 cm^−1^ (Figure 1b and Figure 2b, insets). The appearance and relative intensity of these bands depends on the ND brand. These bands could be surface groups bound to metal species or nitrile [55] or other C–N–C, C–N–N and similar nitrogen-based vibrations [56], which may be the manifestation of N or N vacancy centers in the ND cores [57]. These bands were found in graphene [12] but require more studies in the case of nanodiamonds. The complete elucidation of these groups is more difficult compared to other groups as only DRIFT modality can be used. The comparison of detectors shows that the selection does not significantly affect the band quality in this range, although photovoltaic detection tends to produce unresolved bands.

From the viewpoint of different aggregates in ND samples or different characteristic sized in ND brands, DRIFT measurements should be corrected to radiation reflections from particles of different sizes [54], and a direct comparison with DRIFT may not be very correct, and may require special attention. 

### 4.2. ATR–FTIR

**Figure 9:**
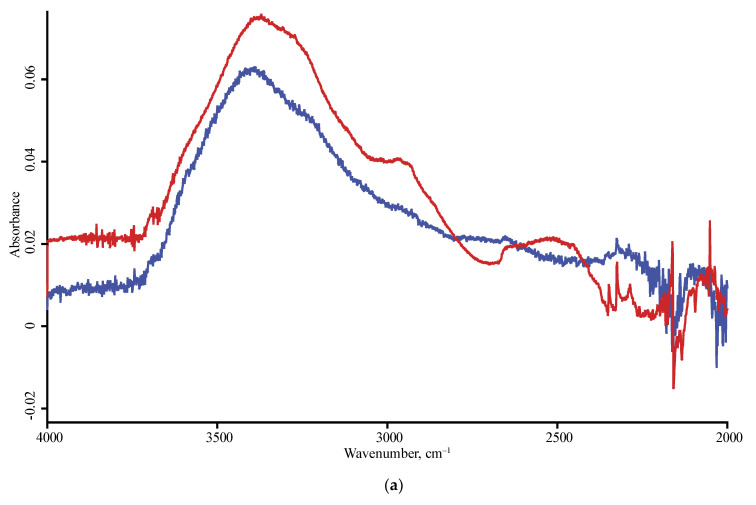

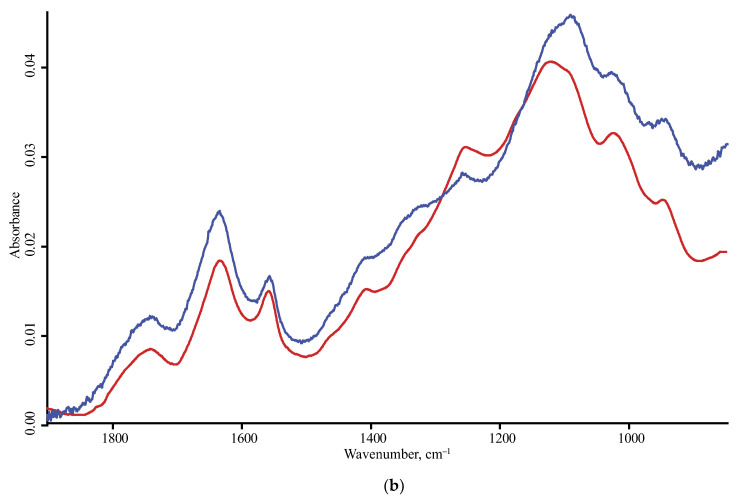
ATR mode, UDA-SP nanodiamonds; red, LN-MCT photovoltaic detector; blue, DLaTGS detector (**a**) 4000–2500 cm^−1^, (**b**) 2000–800 cm^−1^ range. Spectra are not smoothed but normalized to maximize each spectrum.

Due to low sensitivity in the far and Mid-IR range of 4000–2000 cm^−1^, the ATR spectrum shows little-to-no information in this range, CH_x_ bands are only seen for several brands (UDA-GO-SP family). Additionally, in this range, the band of stretching vibrations of –O–H linked by hydrogen bonds, 3690 cm^−1^, is observed only in some brands, and a weaker band at 3715 cm^−1^ is not seen with a DLaTGs detector and is very weak with a photovoltaic detector (Figure 9). Some spectra show a low-intensity sharp artefact band at 2660–2650 cm^−1^ over-imposed with the weak and broad carboxylic O–H stretch visible in some brands only.

Among oxygen-containing groups, the carbonyl C=O stretch (1770–1760 cm^−1^) bands in ATR spectra is the strongest. Compared to DRIFT, the ATR band at 1760 cm^−1^ shows a shoulder band at 1790–1800 cm^−1^, which can be attributed to anhydride or similarly strongly bound carbonyls. This band is weaker than the neighboring band of the water bands at 1630 cm^−1^ and at 1270 cm^−1^, which correspond to carboxyl C–O stretch, and at 1100 cm^−1^ to non-carboxyl C–O stretch. In general, the band at 1770–1760 cm^−1^ is visible, but its intensity is lower than in DRIFT and FTIR–PAS modalities.

Other characteristic bands in ATR spectra are H–O–H bending vibrations (loosely bound, 1643 cm^−1^ and strongly bound, 1620–1635 cm^−1^). All other bands attributed to water in the low-wave Mid-IR range are well pronounced as well. The problem of water-vapor artefact bands at 1580–1500 cm^−1^ is least pronounced in ATR due to the minimum effect of water vapors due to low signal-generating depth; however, the intensity is lower compared to other modalities, and if ATR modality is selected, a high-sensitive detector is required.

Additionally, bands of 1485 cm^−1^ (–CH_2_ wag), at 1390–1370 cm^−1^ (–CH_2_ deformation and C–O–H in-plane bend) are also visible, while the weak band at 1440 cm^−1^, which is attributed to aromatic ring stretch/C–O–H, does not appear. It is noteworthy that many of these bands can be attributed to the different types of functional groups (hydrocarbon and oxygen-containing) and their intensities are much lower than in the case of DRIFT. Along with a lower intensity of carbonyl bands, compared to DRIFT, this may evidence that ATR is more sensitive to the hydrocarbon layers than functional groups. This range is, thus, quite important to be probed with two IR modalities as the ratios. 

ATR becomes the dominant modality for 1100 cm^−1^ and below, when DRIFT shows more enveloping bands and FTIR–PAS shows the saturation effects and high noise levels. The ATR–FTIR spectrum has a weakly pronounced band at 940 cm^−1^ (Figure 3b and Figure 4b), which is not revealed in DRIFT and FTIR–PAS. On the contrary, these two modalities show a band at 960 cm^−1^, but the intensity of 940 cm^−1^ is quite low. In our opinion, this band at 960 cm^−1^ is non-carboxyl out-of-plane C–O–H bend, while the peak at 940 cm^−1^ is =CH_2_ wag, which is revealed in brands with large contents of nonsaturated and aromatic hydrocarbons (UDA-SP, D-G01P brands). The same situation is for the band at 830 cm^−1^ (aromatic CH_2_). A characteristic band at 410 cm^−1^ is clearly visible only in ATR and can be attributed to C–C in-phase vibrations [40]. However, the bands at 760 and 610 cm^−1^ have lower intensities compared to other modalities.

### 4.3. FTIR–PAS

In general, FTIR–PAS spectra at IMF of 1.6 and 2.5 kHz are similar to DRIFT and show the same intensity ratios. For most brands spectra at IMF of 1.6 and 2.5 kHz are the same relative to intensity ratios. In the range 4000–3600 cm^−1^, FTIR–PAS exhibit weak stretching vibration bands of isolated hydrogen bonded species at 3780 cm^−1^ [35], which is not visible with other modalities, and the sensitivity towards other hydrogen-bonded species is similar to DRIFT. Bands at 1750, 1640, 1250, and 1100 cm^−1^ are present in all FTIR–PAS spectra for the whole IMF range 1.6–10 kHz.

Weak bands of asymmetric CH_x_ vibrations in the range 2955–2920 cm^−1^ are pronounced, and the sensitivity is the same as in the case of DRIFT, while the sensitivity towards symmetric vibrations seems lower than in DRIFT. The range 2700–1900 cm^−1^ is noisy, contains the CO_2_ peaks, and are not informative. 

As in ATR, the carbonyl band at 1760 cm^–1^ shows a shoulder at 1790 cm^–1^ of conjugated carboxyls. The bands at 1610 and 1560 cm^−1^, characteristic of some ND brands, are quite intense, as in DRIFT; in this range, FTIR–PAS provides the same resolution (Figure 5). However, in this range, FTIR–PAS experiences the maximum effect from water vapor peaks, so this can lead to a wrong interpretation of the results (because of negative water vapor sharp bands). Thus, DRIFT seems the most expedient in this range. However, the CH_2_ band at 1450 cm^−1^ is almost hidden being in the shoulder of the more intense C–O–H broad band at 1330 cm^−1^.

In an FTIR–PAS spectrum recorded with an interferometer frequency of 2.5 kHz or higher, FTIR–PAS provides a different picture in the 1900–1500 cm^−1^ range compared to DRIFT and ATR. FTIR–PAS shows a different ratio of intensities of major bands of C=O stretch of isolated carboxyl groups at 1760 and a band of HOH vibrations (1635–1620 cm^−1^). While in almost all the brands, the C=O band has the maximum intensity compared to other bands, in FTIR–PAS, this band is higher in intensity only for an IMF 1.6 kHz, while for IMFs of 2.5–10 kHz, the most intense is the water band. 

For the range 1500–1000 cm^−1^, the band at 1100 cm^−1^ (non-carboxyl C–O stretch) is equally as strong as in DRIFT and ATR–FTIR, but FTIR–PAS shows higher sensitivity to bands at 1330 and 1260 cm^−1^ compared to DRIFT, and the shape of the IR spectra in this range is coincident with ATR spectra. The range 1400–1200 cm^−1^ is less resolved compared to ATR but still has a higher sensitivity compared to ATR. The region of 1200–900 cm^−1^ is not well-defined intensity-wise, as the intensities are distorted due to saturation effects [32,58], which was previously shown to have effect on FTIR–PAS identification for soil nanoparticles [30].

Comparison with DRIFT shows almost the same situation as FTIR–PAS to ATR comparison: matrix vibrations mode intensities in DRIFT are degraded, although seen clearly; in concordance with the literature, and much lower sensitivity in the 2500–2000 cm^−1^ region is shown, compared to FTIR–PAS [59].

Contrary to DRIFT, especially under the conditions of rapid-scan modes used in this study, FTIR–PAS spectra are mainly affected with thermal penetration depth, which is governed by thermal parameters of the ND diamond matrix and independent from the particle size. Thus, FTIR–PAS spectra have become directly comparable from the viewpoint of composition being more ready, while retaining most features of DRIFT unrevealed in ATR spectra. In the frames of this study, no significant differences between FTIR–PAS and DRIFT spectra were observed (Figure 5).

### 4.4. ND Brand Features

The aim of this study was not the complete characterization of specific brands, but differences in the composition are obvious can be used as examples of capabilities of different IR modalities in ND analysis.

All the brands differ in the position of the main maximum and the width of the band of carbonyl at 1750 cm^−1^. For most brands, it is located at 1740 cm^−1^, for DNA-TAN and DNA-STP it is located at 1786 cm^−1^, for RUDDM, 1760 cm^−1^, and for PL-D-G-01 and PL-D-G02 it is pure bimodal with maxima at 1780 and 1750 cm^−1^ (Figure 1a and Figure 3c). The reproducibility of this band is highest among the other bands in the spectrum and it is usually the same for different brands of the same manufacturer (DNA-TAN and DNA-STP). For most ND brands, this band is a rather wide continuum with a width of 300 cm^−1^ overlapped with the water peak at 1640 cm^−1^, but its left shoulder is well resolved (Figure 1b, Figure 2b, Figure 3c, and Figure 4c). The shape of this band is not affected by drying (Figure 7 and Figure A3, Appendix A). 

From the viewpoint of ND brand characterization, these groups of nanodiamonds can be distinguished by the amount of bound water (1630 cm^−1^) and the intensity of the OH continuum 3600–3000 cm^−1^ and carboxyl C=O and C–O–H bands at 1760–1740 and 1440–1340 cm^−1^. Upon drying, the spectra of such brands show a decrease in the bands associated with loosely bound water, while the intensities of the carboxyl bands remain the same. For the RUDDM brand, Figure A1 (Appendix A) shows that a decrease in the peak at 3695 cm^−1^ (water hydrogen bonds, there is an increase in the band 3715 cm^−1^ (hydrogen bonds for non-water species). Additionally, these brands do not show a separate band of loosely bound water at 1643 cm^−1^, and this is revealed as a blue-shifter asymmetry of the 1630 cm^−1^ band.

It is noteworthy that the intensities and intensity ratio 1760/1630 cm^−1^ and other peaks in the range 1900–1500 cm^−1^ can probably be used for ND brand selection. The nanodiamonds with high colloidal solubility have higher intensities of both major bands, and usually sharper shapes, which may evidence a domination of single moieties at the surface (carboxyl groups) and larger amounts of bound water. Additionally, the peak at 1760 cm^−1^ is located at 1760–1750 cm^−1^, which corresponds to the existing data on carboxyl carbonyl and is accompanied with bands at 1460–1440 cm^−1^ (carboxyl C–O–H) and larger intensities of –O–H continuum at 2650 cm^−1^. The intensity of the carboxyl band is higher than in the water band. "Insoluble" NDs have a water peak which is more intense than the carboxyl band and is accompanied with weak bands of surface bound water at 1610–1600 cm^−1^; other carboxyl-related bands have lower intensities and other C–O bands are more intense. 

Thus, these two bands can be used for the preliminary estimation of their properties in aqueous solutions, and for monitoring the properties upon thermal or directed chemical treatments. Our studies show that any FTIR modality can be used for this, with ATR having the advantage of working with small amounts and dried dispersions, while DRIFT provides better sensitivity, better distinguishing of the signal from gaseous water artefact peaks. In this task, FTIR–PAS does not have real advantages from the viewpoint of sensitivity and resolution, if other bands in the spectra are not considered.

From the viewpoints of classification, C–H bands provide relevant information with rather different compositions of brands. Most brands, aliphatic CH_2_ (2940–2930 and 2850 cm^−1^) have the maximum intensity of corresponding bands, evidencing the contribution from long aliphatic chains. In several cases, such as RDDM, this correlates with the graphite as the ND production source. However, UDA-GO-SP-M1 and UDA-GO-SP-M2 show predominant peaks at 2950 and 2880 cm^−1^, corresponding to CH_3_ groups, and PL-D-G01P reveals similar intensities of CH_2_ and CH_3_ bands. UDA-GO-SP-M1 and -M2 also show a medium-intensity band at 2970 cm^−1^ of alkene groups. Several brands, PL-D G01P and RUDDM also show high intensity bands over 3000 cm^−1^, assigned to aromatic compounds which, in the latter case, correlate with the manufacturers’ information of the ND source material, trinitrotoluene. This correlates with the bands at 1000–700 cm^−1^ (Table 5), attributed to aromatic C–H vibrations. Brands with high contribution from C–H have distinct bands in near IR DRIFT of 4800–4100 cm^−1^ (Figure 1a and Figure 2a). To sum up, a detailed study of C–H composition of NDs may require both DRIFT and ATR modalities to have the maximum sensitivity in high-frequency and low-frequency parts of the spectra to provide cross checking in different spectral ranges and modalities. However, some caution with this is in order: such bulk spectra relate to nanodiamonds as materials, which can in fact contain ND clusters as well some accompanying components that may seriously affect IR spectra. For instance, ND analysis of visible spectra of some ND brands with visible transmission/optoacoustic spectroscopy with multistage centrifugation reveal the smallest non-diamond sp^2^ fraction [51], which can affect the overall unfractionated sample spectra.

Apart from major bands than can be used for general estimations of ND brands, spectra in different modalities and detectors reveal a more detailed difference in NDs. For instance, PL-D G01P, UDA-S and UDA-SP, and DNA-STP form a specific subset of ND brands of different manufacturers, which have several common features, already discussed above upon modality comparison. They have distinct bands at 1610 and 1560 cm^–1^ that are absent in most other brands (Figure 6). The band at 1560 cm^−1^ is considered the most uncertain for complex samples [29] and can be attributed to carbon bonds [60,61,62] and absorbed water. It is noteworthy that the brands with the distinct band at 1560 cm^−1^ have the main water band red-shifted, compared to the most common position of 1635 cm^−1^. For UDA-GO-SP and PL-D G01P, the ATR spectrum contains only one band of stretching vibrations of the carbonyl group –C=O at 1750 cm^−1^, as with other brands, while in DRIFT and FTIR–PAS, a low-intensity band of deprotonated carboxyl at 1680 cm^−1^ appears [44], which distinguishes this brand from other counterparts, even from the same manufacturer. It is noteworthy that PL-D G01P shows a structure of the band of 1630 cm^–1^ with maxima at 1646, 1636, and 1626 cm^−1^ and a band at 980 cm^−1^ (Figure 6). The brands with the high intensity of CH_x_ bands (UDA-GO-SP-M1 and UDA-GO-SP-M2 and PL-D G02) show an intensity band at 1190 cm^−1^ that can be assigned to C–C(O)–C vibrations.

**Figure A5:**
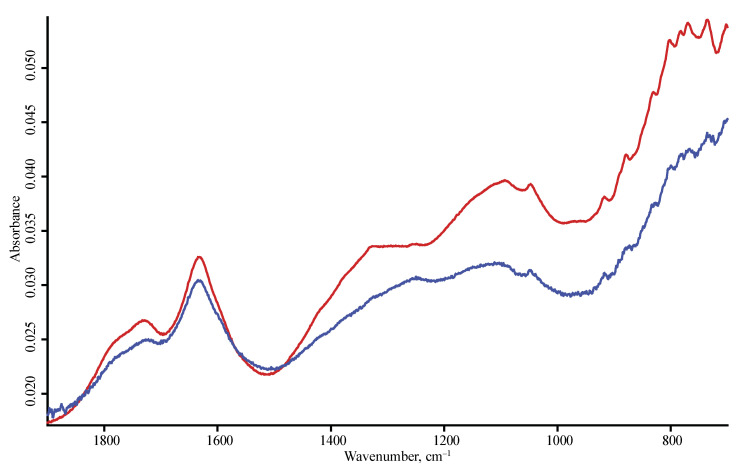
ATR mode, PL-Nanopure-G01P nanodiamonds; red, LN-MCT photovoltaic detector; blue, DLaTGS detector. Spectra are not smoothed but normalized to maximize each spectrum.

From the viewpoint of C–H features, many brands also reveal some specific features. UDA-GO-SP-M1 and UDA-GO-SP-M2 are similar and, apart from the highest intensity of CH_3_ bands among all the studied NDs (Figure A1 and Figure A2, Appendix A), a shoulder band at 1670 cm^−1^ appears (DRIFT), which can be attributed to C=C stretch (Table 5). As well, UDA-GO-SP-M1 and UDA-GO-SP-M2, brands show a distinct band at 830 cm^−1^ (Figure A2c, Appendix A). PL-D-G01 is distinguishable from all other brands by a series of sharp and rather intense bands at 920, 880, 830, 800, 785, 770, and 740 cm^−1^ (Figure A5, Appendix A), which can also be attributable to aromatic chains, as well as the high intensity of the band at 630 cm^−1^. PL-D-G01P shows intense bands at 1480 cm^−1^ (C–C aromatic stretch, Figure 1c) and 3035 cm^−1^ (C–H aromatic stretch, Figure 1b).

As we mentioned in the previous sections, the range 2300–2000 cm^−1^ is characterized with a series of weak and rather sharp bands, which are very characteristic for certain brands. The band at 2230 cm^−1^ is characteristic to UDA-GO-SP, PL-D-G01 P, UDA-TAN, PL-D G01, and the band at 2050 cm^−1^ is present in DRIFT spectra of different brands (Figure 1b and Figure 2b, insets), UDA-GO-SP-M1, UDA-GO-SP-M2, UDA-SP, SDND, and RUDDM. In several cases, this band falls within the left shoulder of the continuum band of the carbonyl, so it is completely undetectable. The band at 2140 cm^−1^ is characteristic for most brands and is the only band in this range for UDA-S, UDA-S-GO, and PL-D-G02.

### 4.5. Modality Comparison

Contrary to some similar studies on IR modality comparison for complex objects [29,30,36,63,64], we cannot conclude that all the modalities are equal in ND characterization and can be used alone. Though it is rather obvious that all the modalities can provide the relevant information on ND surface composition, not a single modality provides the whole information, and the combination of modalities seems quite important. In our opinion, this could result from the DRIFT conditions used in this study: contrary to other studies [29,30,36,63,64], we used a bare mirror as a reference sample, which decreased the noise and provided the good sensitivity and reproducibility. Additionally, using an attachment that provided diffuse-reflection measurements with sample dilution and change provided much more valuable information from DRIFT spectra and made this technique most informative and reliable for ND characterization.

**Figure A6:**
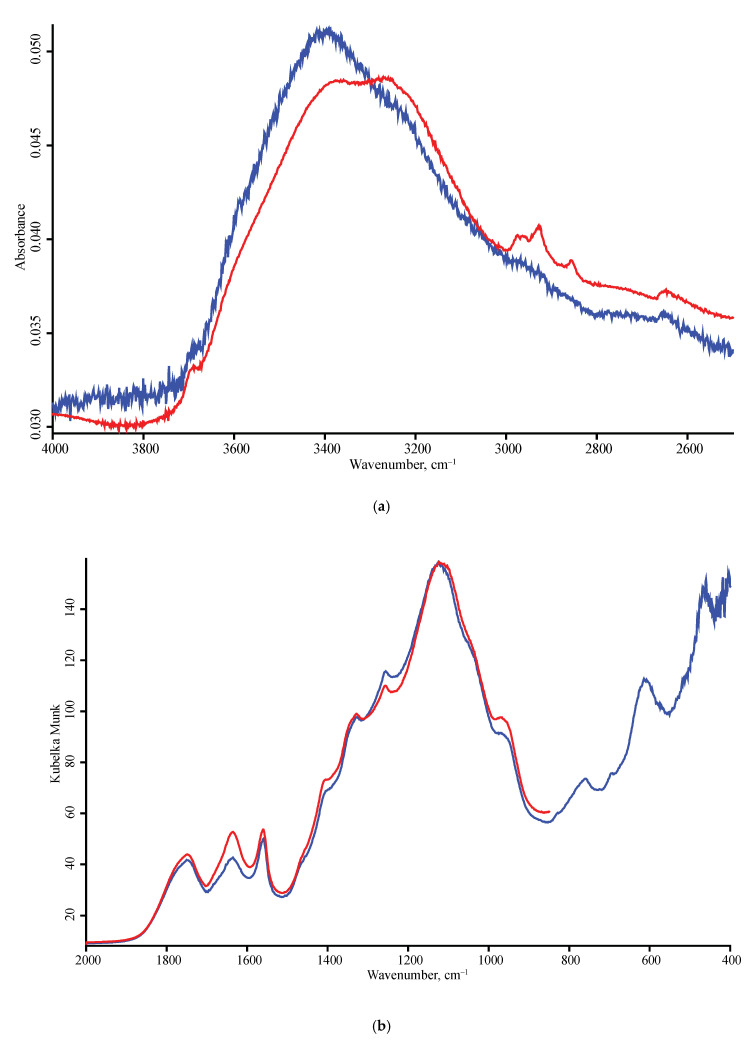
Diffuse reflectance mode (mirror was used for background collection), UDA-SP nanodiamonds; red, LN-MCT photovoltaic detector; blue, DLaTGS detector (**a**) 4000–2000 cm^−1^, (**b**) 2000–400 cm^−1^ range. Spectra were not smoothed but normalized to maximize each spectrum.

Although, in several cases, ATR has the advantage of a small sample amount, DRIFT still seems to be the best choice, as it provides good sensitivity (especially with a photovoltaic detector, Figure A6, Appendix A) and the widest spectral range of near-IR and mid-IR that contains the most important information on NDs. The best sensitivity of DRIFT in hydrogen-bond, CH_x_, and C=O/water ranges makes it possible to distinguish various trademarks, monitor the changes during surface modification and thermal treatment and other studies. The range of 1200–400 cm^−1^ has the secondary value in DRIFT due to saturation and lower sensitivity. High sensitivity of DRIFT provides a rather detailed study in the CH_x_ range and the low-intensity peaks in the 2700–2000 cm^−1^ range that cannot be revealed by other modalities. Still, the cross-examination of ND brands with DRIFT may be taken with caution due to the particle-size effect. From the viewpoint of C–H characterization of ND surface, DRIFT modality has the obvious advantage of sensitivity in the whole mid-IR range, while ATR provides a more unified picture, distinguishing high CH_x_/low CH_x_ brands only; however, it provides additional information due to high sensitivity in the far IR region—e.g., the C–C peaks at 410 cm^−1^.

**Figure 10:**
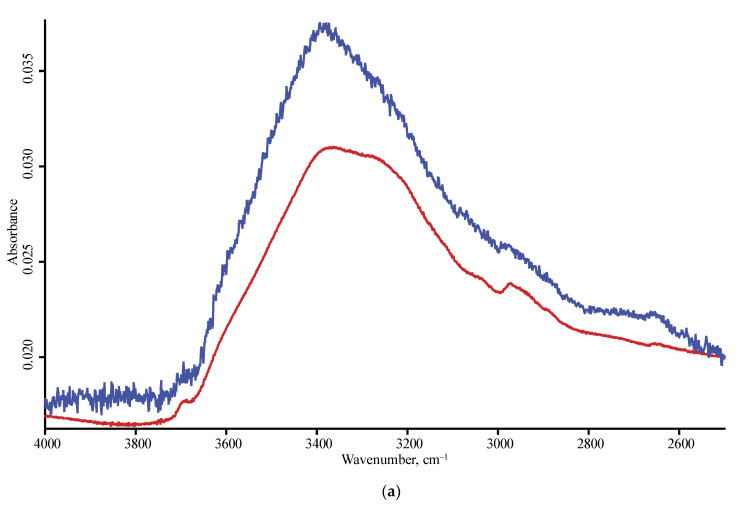

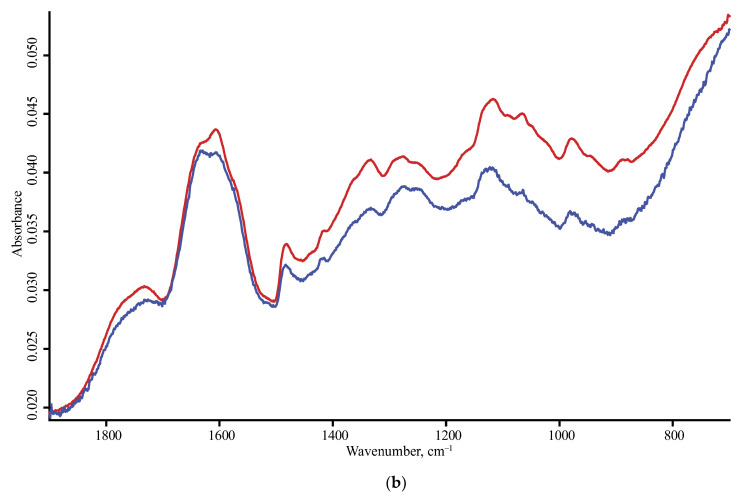
ATR mode, PL-D-G-01P nanodiamonds; red, LN-MCT photovoltaic detector; blue, DLaTGS detector; (**a**) 4000–2500 cm^−1^, (**b**) 2000–800 cm^−1^ range. Spectra were not smoothed but normalized to maximize each spectrum.

**Figure 11:**
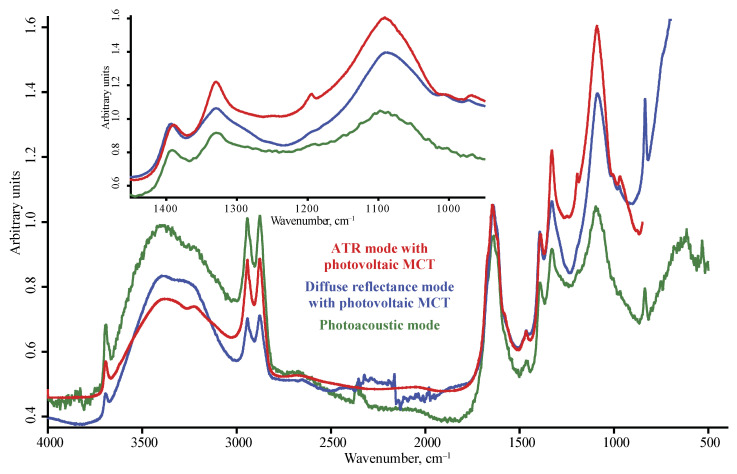
UDA-GO-SP-M1 nanodiamonds; red, ATR mode with a photovoltaic MCT detector; blue, diffuse reflectance mode with a photovoltaic MCT detector; green, photoacoustic mode. Spectra were not smoothed but normalized to maximize each spectrum.

ATR provides good sensitivity for liquid-water and C–H bands in nanodiamond spectra in the range 2000–400 cm^−1^, but shows mediocre sensitivity towards the medium and minor bands that are attributed to carbon–oxygen bands. This makes this modality most expedient for monitoring the drying and surface oxidation of nanodiamonds, as these processes change the major functional groups and water [5], while retaining the reference points of C–H bands. ND brand comparison, which is based on CH_x_ bands at 3000 cm^−1^ and minor oxygen-containing groups using ATR is probably less expedient, and DRIFT or FTIR–PAS should be used instead. ATR can be used as a secondary checking technique with DRIFT due to its higher sensitivity in the 2000–400 cm^−1^ range, lower effects from gaseous water, and better resolution in the ranges (Figure 10), when DRIFT and FTIR–PAS techniques experience optical or thermal saturation. In ATR spectra, the spectral information in the range of 2700–1900 cm^−1^ is lost due to the absorption of the diamond crystal itself and the closeness of the nanodiamond/diamond refractive index. Additionally, in the range 1100–400 cm^−1^, water librations should be taken into account. Additionally, our experiments show that detector sensitivity is quite important in ATR, and a room-temperature DLaTGs detector can be recommended for major band monitoring, while a photovoltaic detector seriously increases the spectral information (Figure 11), making ATR closer to FTIR–PAS, while retaining all the advantages of ATR.

Compared to ATR–FTIR, FTIR–PAS in general provides more informative spectra and shows the maximum sensitivity or resolution or both for absorption bands that do not have enough sensitivity in ATR-IR spectra—the whole continuum of C=O stretch of carboxyl groups (1760 cm^−1^) and a better resolution in a complex band 1500–800 cm^−1^ with C–H, C–C, and C–O. Still, the comparison of FTIR–PAS and DRIFT shows not many differences from the latter with a room-temperature DLaTGs detector, although a lower sensitivity compared to DRIFT was detected with a photovoltaic detector. Along with a higher noise, FTIR–PAS, especially at a single interferometer modulation frequency, cannot be recommended as a primary IR technique for nanodiamonds. Its main feature is a lower dependence on the particle size, due to large signal-penetration depth, resulting from high thermal-conductivity of nanodiamond. Thus, it can be used as a support/check technique for either DRIFT or ATR. Another feature of FTIR–PAS, depth profiling with an IMF change obviously does not affect the spectra in a positive way and can be instead used for investigating thermal properties of nanodiamonds. Additionally, the disadvantage of this modality is a high penetration depth (up to 1 mm), which requires rather large sample volumes lest to have the effect of sample support on the spectra.

Thus, three scenarios can be considered. (1) An overall survey of nanodiamonds using the major bands of CH_x_, water, and carboxyl groups; this may be implemented with any single modality. ATR is preferable from the simplicity and rapidity aspects and does not require a sensitive detector; DRIFT can be used in all the conditions; FTIR–PAS at a low IMF with a careful check for artefacts from gaseous water at 1600–1500 cm^−1^ can be used. (2) Brand comparison by major and secondary bands (main surface moieties) to show the changes upon some surface modification, centrifugation, etc. Such a scenario requires two IR modalities; DRIFT as a primary modality to have the maximum sensitivity of all the bands. ATR can also be used but requires a sensitive detector. The pairs DRIFT/ATR, DRIFT/PAS or ATR/PAS can be used; FTIR–PAS as a primary technique is not recommended due to a high noise level. (3) The case of a complete analysis of the surface required for applications of nanodiamonds, especially in biomedicine. In such a scenario, all the modalities should be used. DRIFT should be used with a high-sensitive detector in the NIR region to cross-check the major C–H and HOH bands and in the range 2700–1900 cm^−1^; while ATR is used at 2000–300 cm^−1^. The range 1500–900 cm^−1^ is measured with DRIFT, ATR, and FTIR–PAS (probably at two IMFs) to reveal possible artefact peaks, optical/thermal saturation, and revealing the nature of complex peaks can be comprised by hydrocarbon and functional groups).

Thus, the region of 1500–900 cm^−1^ should be further taken into account as the selected unmodified ND brands did not allow for the final reliable information on the bands at 1470, 1440, 1400, 1370, 1330, 1270, 1210, 1190, 1140, 1100, 1050, 950 cm^−1^; this would require a more detailed study with samples with known functional groups and a cross-comparison with other nanoparticle materials different from nanodiamonds.

Apart from the band maxima positions and relative intensities, another parameter of bands is its width is peak widths, which can be related to the crystal structures or grain sizes of nanodiamond materials. From the first estimations, the band width for most ND samples within a single modality and measurement conditions differed insignificantly, though some changes in the same samples for different modalities can be elucidated. However, the detailed analysis of the peak width, considering the ND characteristic size and modality-based factor, requires modeling that was out of the scope of this study.

Although it is not a direct comparison of IR modalities for NDs, it is noteworthy that CH_x_ bands at 3000–2800 and 1300 cm^−1^ (shoulder) of stretching and bending CH_x_ vibrations, respectively, do not disappear or change significantly upon drying, oxidizing by rather harsh conditions of acidic treatments [5] so they can be used as a kind of internal standard for quantification and brand comparison. 

From the viewpoint of quantification, which was not considered in this study, some conclusions can be given: in general, most obvious application is the estimation of water and carbonyl contents aby the area 1900–1200 cm^−1^. In this case, all the techniques showed enough sensitivity for the estimation and discrimination of various samples. Both ATR and DRIFT seem most expedient due to a low noise level and sensitivity. In the case of hydrocarbon contents, DRIFT shows the maximum possibility, especially with a photovoltaic detector due to high sensitivity and a satisfactory noise level. The identification shows that DRIFT can potentially be used for the discrimination of CH3 and alkane/alkene/aromatic CH_x_ groups. This would require a reference method, such as NMR, the elucidation of absorption coefficients for the ND materials of these bands, and working out the data handling for the quantification (most probably, integrated peak intensities should be used as was shown for soil particles [30]). Among other possible quantification techniques are correlation studies between several counterparts of the functional groups like carboxyls at 2700, 1800, and 1400 cm^−1^), studies of possible contributions of core groups at 2400–2200 cm^−1^ and quantification of “non-standard” groups at 1600–1300 cm^−1^. In all these studies, DRIFT with a high-sensitive detector is most expedient. The range 1100–400 cm^−1^, though it contains some important information on NDs, seems most troublesome from the viewpoint of quantification, due to possible optical and thermal-saturation effect, possible contributions of several functional groups and rather high noise levels in spectra. This requires some further studies before discussing the real possibilities. From the viewpoint of this study, the use of FTIR–PAS for ND surface quantification seems less expedient compared to ATR and especially to DRIFT. 

## 5. Conclusions

Thus, from the viewpoint of qualitative analysis of the nanodiamond surface by FTIR spectrometry, we cannot conclude that all the modalities are equal in ND characterization and can be used alone. In fact, the analysis of the ND surface requires all three modalities—diffuse-reflectance, attenuated total reflection, and photoacoustic modalities. Although ATR is advantageous from the viewpoint of small sample amounts, DRIFT seems to be the best choice as it provides good sensitivity, especially with a high-sensitivity photovoltaic detector and, thus, results in the maximum number of bands among all three modalities. Additionally, the configuration of DRIFT measurements used in this study provided low noise and good reproducibility of measurements, which is relevant for quantification, which should be the subject of a separate study. As discussed, three scenarios can be considered for the use of FTIR modalities for nanodiamonds: (1) An overall survey of nanodiamonds using the major bands of CH_x_, water, and carboxyl groups; it may be implemented with any single modality. Here, ATR is preferable as a simple rapid mode without a need for a sensitive detector; DRIFT can be used as well. (2) The second scenario is nanodiamond brand comparison by changes in surface moieties and groups upon some surface modification, centrifugation, etc. This requires two IR modalities—DRIFT to attain the maximum sensitivity, and ATR as a secondary technique (FTIR–PAS is not recommended as a primary technique due to a high noise). (3) The complete analysis of the surface. Here, all three modalities should be used—DRIFT with a high-sensitive detector and in the NIR region to cross-check the major bands in the region 2700–1900 cm^−1^ and ATR at 2000–300 cm^−1^. The most informative range of 1500–900 cm^−1^ should be measured with all three modalities to check for possible artefact bands, saturation effects etc. to ensure the reliability of qualitative and quantitative analysis.

## Figures and Tables

**Table 1:** Nanodiamonds used in the survey.

Product Name	Description	Manufacturer
RDDM	detonation polycrystalline diamond of RDDM grade, fraction 0–0.125	“Real-Dzerzhinsk” Ltd., Dzerzhinsk, Russia
RUDDM a	nanodiamond material of RUDDM grade, fraction 0–150	
SDND	5 wt. % Single-Digit NanoDiamonds aqueous suspension	PlasmaChem GmbH, Berlin, Germany
PL-D-G	Purified powder grade G	
PL-D-G02	Extra-pure, grade G-02	
PL-D-G01P	agglomerate free, positively charged	
PL-Nanopure-G01P	4 wt.% nanodiamonds aqueous suspension, grade G	
DNA-TAN	DNA-TAN	Special Construction-Technological Bureau “Tekhnolog”, St. Petersburg, Russia
DNA-STP	DNA-STP	
UDA-S	UDA-S, ultradispersed diamond powder	The Laboratory of ultradispersed diamonds of Joint Stock Company Federal Research and Production Center ALTAI, Biysk, Russia
UDA-S-GO	UDA-S-GO, ultradipersed diamond powder of deep purification	
UDA-SP	UDA-SP, ultradispersed diamonds	JSC “SINTA”, Minsk, Republic of Belarus
UDA-GO-SP	UDA-GO-SP, deep purified ultradispersed diamonds	
UDA-GO-SP-M1	UDA-GO-SP-M1, modified ultradispersed diamonds, type M1	
UDA-GO-SP-M2	UDA-GO-SP-M2, modified ultradispersed diamonds, type M2	

**Table 2:** Parameters of recording NDs spectra in the middle IR by FTIR–PAS.

Spectral range, cm^−1^	4000–500
Resolution, cm^−1^	4
Background scan	64; 256
Sample scan	64; 256
Phase resolution	10
Phase correction mode	Mertz
Zero filling factor	2
Apodization function	Blackman–Harris 3-Term
Aperture setting	8 mm
Interferometer frequency	1.6; 2.5; 5 kHz
Sample and background pre-amplification gain	B (middle amplification)
Sample signal gain	Auto
Detector	microphone
Source	MIR
Beam splitter	KBr

**Table 3:** Parameters of recording NDs spectra in the middle IR by ATR–FTIR.

Spectral range, cm^−1^	4000–370 (with DLaTGS detector) or 6000–700 (with MCT detector)
Resolution, cm^−1^	2
Background scan	128
Sample scan	128
Aperture setting	8 mm
Phase resolution	4
Phase correction mode	Mertz
Zero filling factor	1
Apodization function	Blackman–Harris 3-Term
Sample and background pre-amplification gain	“Ref” (without amplification)
Background signal gain	Auto
Sample signal gain	Auto
Scanner velocity	10 kHz
Detector	Room temperature DLaTGS or liquid nitrogen cooled photovoltaic MCT
Source	MIR
Beam splitter	KBr
Background	Diamond crystal with a lowered pressure screw with a flat end

**Table 4:** Parameters of recording ND spectra in the near to middle IR by DRIFT.

Spectral range, cm^−1^	7000–400
Resolution, cm^−1^	2
Background scan	256
Sample scan	256
Phase resolution	16
Phase correction mode	Mertz
Zero filling factor	2
Apodization function	Blackman–Harris 3-Term
Aperture setting	3 mm
Sample and background pre-amplification gain	“Ref” (without amplification) for DLaTGS detector A (standard amplification) for MCT detector
Background signal gain	Auto
Sample signal gain	Auto
Scanner velocity	10 kHz
Detector	Room temperature DLaTGS or liquid nitrogen cooled photovoltaic MCT
Source	MIR
Beam splitter	KBr
Background	Mirror

**Table 5:** The assignment of major bands common to most ND brands [16,34,35,36,37,38,39,40].

Wavenumber	Assignment	ATR	DRIFT	PAS *
5900–5600	2ν C–H aliphatic chain stretching	—	W	—
5300	Water combination band *av*_1_ + *v*_2_ + *bv*_3_; *a* + *b* = 1	—	Mb	Wb
4800	Aromatic C–H combination bands (?) **	—	Wb	—
4500–4100	Aliphatic C–H combination bands	—	Wb To Mb	W
3715	Hydrogen-bonded –O–H···H–O– stretch	—	Wp	Wp
3695	Hydrogen-bonded –O–H···H_2_O stretch	—	Mp	Wp (noisy)
3569	Hydrogen-bonded RO–H···H_2_O H–OR stretch (?)	—	W	W (noisy)
3450–3420	Liquid: antisynchronous stretch *v*_3_	Mv	Sv	Mv
3407	O–H stretch and intermolecular hydrogen bonds (unresolved)	Mb to Wb	—	Mb to Wb
3290	H–O–H bend of liquid adsorbed water, 2*v*_2_	—	Wv	W
3230–3210	Liquid: synchronous stretch, *v*_1_	Sv	Sv	Sv
3050	Aromatic C–H stretching	W to none	S	M
2970	Alkene C–H stretch	W to none	M	W
2950–2940	Aliphatic C–H, CH_3_ antisymmetric stretch	W to none	S	M
2940–2930	Aliphatic C–H, CH_2_ antisymmetric stretch	—	S	S
2880–2870	Aliphatic C–H, CH_3_ symmetric stretch	W to none	S	M
2850–2835	Aliphatic C–H, CH_2_ symmetric stretch	W to none	Wb	M
2750–2550	Carboxylic O–H stretch	Wb to none	Mb to Wb	Wb to none*
2150	Water combination band *v*_2_ + *L*_2_	Wb	Wb	Wb
1800–1780	C=O stretch of conjugated carboxyl groups	M	S	S to M *
1765–1730	C=O stretch of monomeric carboxyl groups	Mb	Sb	Sb to Mb *
1670	C=O stretch of non-carboxyl carbonyl (?) C=C stretch	W	W	M
1644–1642	H–O–H bend of liquid water, *v*_2_	Sv	Mv	Mv
1630–1625	H–O–H bend of adsorbed liquid water, *v*_2_	Sv	Mv	Mv
1610	H–O–H bend of adsorbed liquid water, *v*_2_	Ssh	Msh	Msh
1580–1560	adsorbed water, C=C stretch, (?)	M	M	M
1470–1450	sp^3^ CH_2_ wagging	S	M	
1440	Carboxyl C–O–H in-plane bend Aromatic, ring C=C stretch	—	Wb	W
1410	Carboxyl C–O–H in-plane bend	Msh	Msh	Msh
1400–1395	Non-carboxyl C–O–H in-plane bend CH_2_ deformation (scissors)	Msh	Ssh	Ssh
1373	Non-carboxyl C–O–H in-plane bend CH_3_ deformation (umbrella)	M	Ssh	M
1330	Non-carboxyl C–O–H in-plane bend (?)	S	M	S to M *
1270–1267	Carboxyl C–O stretch	W	M	M
1245–1235	C–N stretch	W	W	W
1192	C–C–C (?)	—	M	W
1145–1130	C–O–C (?)	—	M	W
1103	Non-carboxyl C–O stretch	Sb	Sb	Sb
1060–1040	In plane –C–H bend (non-aromatic) and carbohydrates (?)	W	Wsh	Wb
1000–500	Water librations, *L*_2_	Sb	Sb	Mb to Wb
960–940	Carboxyl out-of-plane C–O–H bend, =CH_2_ wagging (?)	M	Wb	M to W *
830	Aromatic =C–H bend	Mp	Mp	Mp
760	Polyaromatic =C–H bend (?)	—	W	M to none *
610	Non-carboxyl out-of-plane C–O–H bend	W	M	Wb to none *
410	(?) C–C in-phase vibrations	M	Wb	—

Notation: S = strong; M = medium; W = weak; p = peak (sharp); b = broad; v = variable; sh = shoulder; * Intensity depends on the modulation frequency (PAS); ** (?) not fully clear.

**Table 6:** Relative standard deviation of band integral intensities in the wavenumber range 3000–2000 cm^–1^ by ATR–FTIR (RDDM ND brand).

Band Center, cm^−1^	High-Wave Boundary, cm^−1^	Low-Wave Boundary, cm^−1^	RSD
2935	2952	2918	0.27
2837	2857	2817	0.25
2650	2673	2626	0.43
1750	1815	1684	0.21
1630	1668	1535	0.18
1400	1417	1393	0.20
1270	1341	1249	0.21

**Table 7:** Relative standard deviation of band integral intensities in the wavenumber range 3000–2000 cm^−1^ by FTIR–PAS (RDDM ND brand).

Band Center, cm^−1^	High-Wave Boundary, cm^−1^	Low-Wave Boundary, cm^−1^	RSD
2935	2952	2918	0.40
2837	2857	2817	0.40
2650	2677	2622	0.27
1750	1852	1691	0.33
1630	1690	1594	0.32
1400	1417	1393	0.33
1270	1341	1244	0.39

**Table 8:** Relative standard deviation of band integral intensities in the wavenumber range 3000–2000 cm^−1^ by DRIFT (RDDM ND brand).

Band Center, cm^−1^	High-Wave Boundary, cm^−1^	Low-Wave Boundary, cm^−1^	RSD
2935	2952	2918	0.34
2837	2857	2817	0.37
2650	2676	2619	0.23
1750	1852	1691	0.10
1630	1690	1594	0.08
1400	1417	1393	0.11
1270	1341	1253	0.11
